# An Unbiased Molecular Characterization of Peripartum Cardiomyopathy Hearts Identifies Mast Cell Chymase as a New Diagnostic Candidate

**DOI:** 10.1016/j.mcpro.2026.101510

**Published:** 2026-01-13

**Authors:** J.F. Mulvey, C. Sailer, J.S. Achter, G.N. Milburn, R.C. Bretherton, K. Kahnert, S. Erbil Bilir, H. Hvid, C. Pyke, F. Gustafsson, L. Adamo, K.S. Campbell, K.M. Herum, A. Lundby

**Affiliations:** 1 Department of Biomedical Sciences, Faculty of Health and Medical Sciences, University of Copenhagen, Denmark; 2 Division of Cardiovascular Biomedicine, University of Kentucky, Lexington, Kentucky, USA; 3 Research and Early Development, Novo Nordisk A/S, Måløv, Denmark; 4 Department of Cardiology, Rigshospitalet, Copenhagen, Denmark; 5 Department of Clinical Medicine, Faculty of Health and Medical Sciences, University of Copenhagen, Denmark; 6 Division of Cardiology, Department of Medicine, Johns Hopkins University School of Medicine, Baltimore, Maryland, USA

**Keywords:** peripartum cardiomyopathy, proteomics, spatial transcriptomics, single nucleus transcriptomics

## Abstract

Peripartum cardiomyopathy (PPCM) is a rare form of acute heart failure that develops in women toward the end of pregnancy or early postpartum. No effective, specific treatment for PPCM is available and heart transplantation or mechanical circulatory support may be required in severe cases where drug treatment for heart failure is insufficient. The mechanisms through which the disease progresses are not well understood, and despite similar clinical characteristics to dilated cardiomyopathy of other etiologies (nonperipartum cardiomyopathy; NPCM) it is not known how the molecular remodeling differs between these groups. We aimed to provide insight into the human PPCM heart using unbiased methodologies, and to use changes occurring within the heart tissue to facilitate biomarker discovery. We obtained heart tissue from female patients with end-stage disease receiving either heart transplantation or left ventricular assist device implantation, or from organ donors without heart disease as a control group. We performed deep proteomics, single nucleus transcriptomics and spatial transcriptomics, providing a comprehensive map of the molecular phenotype in advanced PPCM compared to both control and NPCM hearts. Consistent with similarities in the clinical phenotypes of PPCM and NPCM, we observed regulation of canonical markers of end-stage heart failure in both PPCM and NPCM hearts in comparison to controls. Among the changes specific to PPCM and that were consistently observed across multiple data types and cohorts was an upregulation of chymase and carboxypeptidase A3, consistent with mast cell proliferation/activation. Analysis of the proteome of peripheral blood serum from a larger cohort of patients with PPCM and controls showed that chymase was strongly predictive of cardiomyopathy in peripartum women. PPCM heart tissue is characterized by increased mast cell proteins chymase and carboxypeptidase A3. Chymase may have clinical utility as a biomarker for the diagnosis of cardiomyopathy in peripartum women.

## Highlights

• Peripartum cardiomyopathy heart tissue: proteomics, snRNAseq & spatial RNAseq.
• Mast cell proteins CMA1 & CPA3 specifically upregulated in peripartum cardiomyopathy.
• Validation using bulk RNAseq from a published external cohort.
• Mast cells spatially localize in proximity to cardiac fibroblasts.
• Superior diagnostic performance of chymase compared to NT-proBNP in blood serum.

## Graphical abstract

**Figure:**
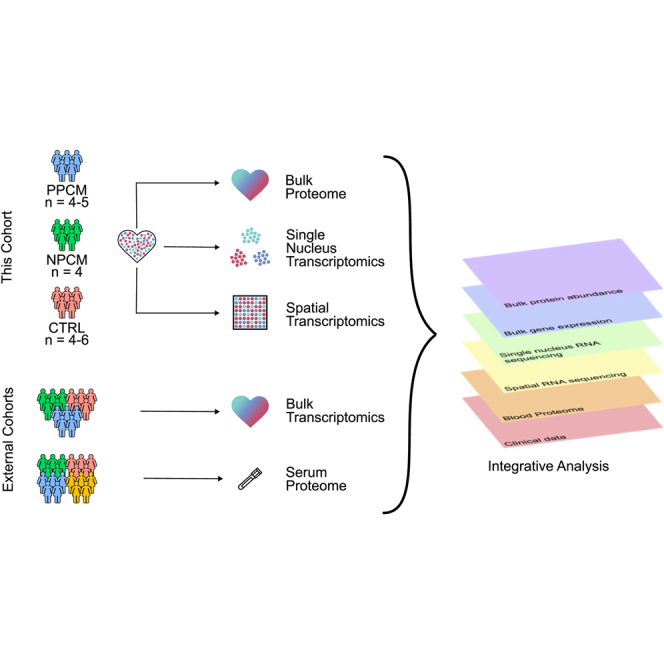

## In Brief

Peripartum cardiomyopathy (PPCM) is a rare and poorly understood form of pregnancy-associated heart failure. Using deep proteomics alongside single-nucleus and spatial transcriptomics, we mapped the molecular landscape of human PPCM hearts and found mast cell proteins chymase and carboxypeptidase A3 uniquely elevated compared to nonperipartum cardiomyopathies. Independent blood serum analyses confirmed chymase as a predictor of PPCM, underscoring its potential as a diagnostic biomarker for peripartum cardiomyopathy.

Peripartum cardiomyopathy (PPCM) is a rare but important cause of heart failure with reduced left ventricular ejection fraction (HFrEF) that presents in the last month of pregnancy or up to 5 months after birth where no other cause is evident (1). Outcome in PPCM is highly variable: some women recover completely with conventional HFrEF drug therapy, some live with persistent heart failure and others deteriorate rapidly to ultimately require heart transplantation or mechanical circulatory support (2). As with other forms of heart failure there is an unmet need for effective therapies.

Diagnosis is also a challenge for the clinician faced with a potential PPCM patient, especially given that there is a large overlap of symptoms between PPCM, normal gestation and preeclampsia—which is itself a risk factor for PPCM (3). This is especially important since early diagnosis has been correlated with better prognosis (4).

We hypothesize that a better understanding of the molecular mechanisms underlying the pathology of PPCM will enable the development of both novel diagnostic and therapeutic strategies. Here, we exploit high-resolution proteomics, single-nucleus transcriptomics, and spatial transcriptomics to outline the molecular and cellular remodeling of human hearts with PPCM in comparison to controls without a history of heart disease, and to characterize where and how this differs from nonperipartum cardiomyopathy (NPCM). We then analyzed these changes in relation to previously published work in independent cohorts to ensure relevance to the PPCM population beyond our own experiments.

## Experimental Procedures

### Collection of Human Cardiac Tissue

Midwall myocardial tissue was collected from either organ donors or from patients with (PPCM or nonischemic dilated cardiomyopathy of any other etiology (NPCM) receiving a heart transplant or implantation of a left ventricular assist device as described previously (5). All procedures were approved by The University of Kentucky Institutional Review Board and informed consent (IRB #46103) was given by patients or their legally authorized representatives. Subsequent analyses of human samples are pursued under ethical licenses H-19088472 and H-22019021 from the National Videnskabsetisk Komité, Regional Ethics Committee, in Copenhagen, Denmark. The studies were conducted in accordance with legislation and requirements set by the Danish National Committee on Health Research Ethics (NVK) and the Medical Research Ethics Committees (MREC) and abide by the principles set out in the Declaration of Helsinki.

### Proteomics

#### Tissue Homogenization, Protein Digestion, and Peptide Desalting

Preparation of heart tissue for proteomics was performed essentially as described previously (6). In short, around 3 mm^2^ of frozen heart tissue from each patient was homogenized in reinforced tubes containing ceramic beads (3 × 1.4 mm, 2 × 2.8 mm) and 200 μL of ice cold lysis buffer (50 mM Tris–HCl pH 8.5, 5 mM EDTA, 150 mM NaCl, 10 mM KCl, 1% Triton-X 100, 1 complete inhibitor cocktail tablet (Roche)) using two cycles of 20 s at 6500 rpm and one additional cycle of 25 s at 5500 rpm (Precellys 24, Bertin Technologies). Lysates were incubated rotating head-over-tail for 2 h at 4 °C followed by centrifugation at 10,000*g* at 4 °C for 10 min. Protein concentration of the supernatant was determined by using Pierce BCA Protein Assay Kit (Thermo Fisher Scientific). One milligram of protein from each sample was precipitated by addition of four volumes of cold acetone (−20 °C), incubated for 1 h at −20 °C, and subsequently centrifugated at 2000*g*, 4 °C for 5 min.

Precipitated proteins were resuspended in 6M guanidine–HCl 50 mM Tris pH 8.5, reduced with 5 mM tris (2-carboxyethil) phosphine (TCEP, Sigma-Aldrich) for 10 min at 95 ˚C and alkylated with 10 mM chloroacetamide (CAA, Sigma-Aldrich) for 15 min at room temperature (RT) in the dark. Samples were then diluted to 2 M guanidine–HCl using 50 mM Tris pH 8.5 and digested by 1:50 (w/w) Lys-C (Sequencing grade, Wako) for 1 h at 25 °C and 700 rpm. Proteins were further diluted to 0.5 M guanidine–HCl with 50 mM Tris pH 8.5 and digested with trypsin (Sequencing grade, Promega) 1:100 (w/w) over night at 37 °C and 700 rpm. Digestion was quenched by addition of trifluoroacetic acid (TFA) to a final concentration of 0.5% (pH ∼2). Digested peptides were desalted by a solid phase extraction system (C18 SepPak, 1 cc, 50 mg, Waters Corp; wash solvent 0.1% TFA or 0.1% formic acid). Peptides were eluted stepwise with 40% acetonitrile (ACN) and 60% ACN and dried (Eppendorf, Concentrator Plus).

#### Tandem Mass Tag Labeling

Tandem mass tag (TMT) labeling was performed using a standard protocol, as described by Zecha et al (7). Dried peptides were resuspended in 50 mM Hepes pH 8.5 and peptide concentration was determined (Lunatic, Unchained Labs). Hundred microgram peptides of each sample were labeled with 100 μg of TMTpro reagents resuspended in 5 μL 100% anhydrous ACN. The final reaction conditions were as follows: TMT-to-peptide ratio 1:1, 4 μg/μl peptide concentration, 20% ACN, 50 mM Hepes pH 8.0. The TMT-peptide mixture was incubated for 1 h at 22 °C and 1000 rpm, and the labeling reaction was terminated by addition of 5% hydroxylamine to a final concentration of 1% for 15 min at 22 °C and 1000 rpm. Samples were combined, acidified to pH 2 with 1% TFA, and desalted on C18 Sep-Pak as described above.

#### Fractionation at High pH

Peptides were resuspended in 0.1% FA and the final peptide concentration was determined (Lunatic, unchained labs). Twenty micrograms TMT-labeled peptides (concentration 1 μg/μl) were fractionated using a PepSep C18 column (120 Å, 1.9 μm, 250 μm × 30 cm, Bruker) on an EASY-nLC 1200 system (Thermo Fisher Scientific) operating at a flow rate of 1.5 μl/min with two buffer lines (Buffer A: 10 mM triethylammonium bicarbonate pH 8, Buffer B: 80% ACN, 10 mM triethylammonium bicarbonate pH 8). Peptides were separated over a 100 min gradient (3–40% B in 57 min, 40–60% B in 5 min, 40–95% B in 10 min, 95% B for 10 min, 95–3% B in 10 min, 3% B for 8 min) and eluent was collected sequentially at 30 s intervals into a total of 24 fractions. Fractionated peptides were acidified with formic acid to a final concentration of approximately 0.1% and dried (Eppendorf, Concentrator Plus). Prior to liquid chromatography tandem mass spectrometry (LC-MS/MS) analysis, peptides were resuspended in 10 μL 2% ACN and 0.1% TFA.

#### LC-MS/MS Analysis

The resulting 24 fractions of TMT-labeled peptides were analyzed on an Orbitrap Eclipse Tribrid mass spectrometer coupled to a Vanquish Neo UPHLC system (Thermo Fisher Scientific). Peptides were separated on a 200 cm μPAC column (Gen 1) at a flow rate of 300 nl/min over a 165 min multistep linear gradient (Buffer A: 0.1% formic acid, Buffer B: 0.1% formic acid, and 80% ACN); 1–5% B in 1 min, 5–10% B in 9 min, 10–30% B in 90 min, 30–50% B in 15 min, 50–97.5% B in 5 min, 97.5% B for 5 min, 97.5–1% B in 5 min and 1% B for 35 min). Column effluent was directly ionized in a nano-electrospray ionization source operated in positive ionization mode and electrosprayed into the mass spectrometer. Spray voltage was set to 2.2 kV, funnel RF level at 30%, and the transfer tube temperature was maintained at 275 °C. Full scan mass spectra were acquired in the orbitrap at a resolution of 120,000, a scan range of 400 to 1400 m/z and an AGC target of 100% or a maximum injection time of 50 ms. Most intense precursor ions (intensity ≥5.0 × 10^4^) with charge states 2 to 6 and monoisotopic peak determination set to “peptide” were selected for MS/MS fragmentation by higher-energy collisional dissociation at 35% collision energy in a data-dependent mode with a 3 s cycle time. The MS2 isolation width was set to 0.7 m/z and the duration for dynamic exclusion was set to 45 s. MS/MS spectra of fragment ions were acquired in the Orbitrap at a resolution of 50,000 and a scan range of 110 to 2000 m/z (AGC target 200%, maximum fill time of 120 ms). All data were acquired without FAIMS Pro Interface.

#### Protein Quantification

Thermo raw files were converted to mzML format using ProteoWizard’s MSConvert (v3.0.23006) tool (8) and analyzed with FragPipe v18.0. (MSFragger v3.5) (9) and Philosopher version 4.3.0 (10) using the built-in “TMT16” workflow with default MSFragger settings. Fixed modifications included carbamidomethylation of cysteine residues and TMTpro labeling of lysine residues, while variable modifications comprised methionine oxidation, TMTpro labeling of peptide N termini, and protein N-terminal acetylation. A maximum of three variable modifications and up to two missed tryptic cleavages were allowed. Spectra were matched against a human fasta database (UP000005640, downloaded 2022/06/07) supplemented with contaminants and decoys (40,840 total entries; 50% decoys, 96 contaminants). Precursor and fragment mass tolerances were set to ±20 ppm. MSFragger search results were validated using Percolator (11) with Philosopher handling protein inference and false discovery rate (FDR) filtering, applying a 1% FDR at the PSM, peptide, and protein level. For quantification, TMT-Integrator was configured with a precursor intensity fraction threshold of 0.75, retaining only unique peptides. Summed MS2 reporter ion intensities were employed for ratio-to-abundance conversion. Peptide-spectrum-matches were aggregated to the gene level, logarithmically (base 2) transformed and used as input for downstream processing in R/python.

Using the pandas library in python (version 3.10), quantified proteins were filtered for identifications with two or more peptide spectrum matches, and samples were normalized by subtraction of the intensity of the median protein from all values in that sample. Proteins with missing values were removed from the dataset. Differential abundance was calculated using an empirical Bayes-moderated F-test or *t* test as indicated, with the experimental group as the sole independent variable (12).

### Single Nucleus Transcriptomics

#### Nuclei Isolation

Total RNA were obtained from flash-frozen tissues using a previously described protocol (13). Tissues were homogenized in QIAzol lysis reagent (Qiagen) using a Precellys device. RNA molecules were isolated in phase gradients and then purified with isopropyl alcohol and ethanol precipitations. RNA integrities were assessed using a TapeStation device (Agilent; Supplemental Fig. S3*B*). Tissues from the same experimental group were pooled for downstream processing.

Single nuclei were obtained from flash frozen donor heart tissues using mechanical stress as previously described (14). Pooled tissue samples were first powdered in a mortar on dry ice. Then they were homogenized using a 7 ml dounce homogenizer (Sigma-Aldrich) in homogenization buffer (250 mM sucrose, 25 mM KCl, 5 mM MgCl2, 10 mM Tris–HCl, 1 mM dithiothreitol (DTT), 1x protease inhibitor, 0.4 U/μl RNaseIn (Invitrogen), 0.2 U/μl SUPERase In (Invitrogen), 0.1% Triton X-100 in nuclease free water) with eight loose strokes with pestle A followed by eight tight strokes with pestle B. Homogenate was filtered through 40 μm cell strainer (pluriSelect) and centrifuged (500g, 5 min, 4 °C) to obtain nuclei pellet. The supernatant was removed and the nuclei pellet was resuspended in storage buffer (1x PBS -Mg/Ca), 1% bovine serum albumin, 0.2 U/μl Protector RNaseIn (Roche). Isolated nuclei were stained with NucBlue Live ready Probes reagent (Thermo Fisher Scientific) and nuclei integrities were checked under a fluorescent microscope (Supplemental Fig. S3*C*). Hoechst 33342 positive nuclei were purified by fluorescent-activated cell sorting (BD FACSymphony S6, nozzle Size: 70 μm, amplitude: 8.1 V, frequency: 87 kHz) (Supplemental Fig. S3, *E*–*F*). Nuclei purity was then checked again under a fluorescent microscope (Supplemental Fig. S3*D*). Purified nuclei were further processed with 10X Genomics Chromium Next GEM Single Cell 3′ Kit according to manufacturer instructions.

#### Chromium 10X Library Preparation and Sequencing

Nuclei were counted automatically by using a NucleoCounter NC-202 (ChemoMetec) and a Countess II (Life Technologies) at two separate counts and loaded on the Chromium Controller (10X Genomics) with a targeted nuclei recovery of 10.000 per sample. In addition, 3′ complementary DNA (cDNA) libraries were prepared using 10X genomics Chromium Next GEM Single Cell 3′ Reagent Kits v3.1 (Dual Index) according to the manufacturer’s instructions. Quality control of cDNA and final libraries were done using a TapeStation System (Agilent). Libraries were quantified using Kapa Library Quantification Kit (Roche) and were pooled for sequencing. Pooled cDNA libraries were sequenced using a NovaSeq platform (Illumina) with a mean read depth of 59,304 read pairs per nucleus.

#### Analysis of Sequencing Data

CellRanger (7.0.1, 10x Genomics) was used to demultiplex BCL files and count expression, with intronic reads included but otherwise default parameters. The human reference file was obtained from 10x (version 2020-A (July 7, 2020)).

Ambient RNA was removed using cellbender (15), and then Demuxafy was used to both remove droplets containing multiple nuclei and demultiplex our pooled samples into the individual patient samples (16). Both heterotypic (containing common genetic variants from more than one individual) and homotypic doublets (by majority vote of Freemuxlet (17), Souporcell (18), Vireo (19), scDblFinder (20)) were excluded from further analysis.

Seurat 4.3 (21) was used for quality control and downstream analyses. Droplets with data pertaining to <800 transcripts, <500 genes, or >20% transcripts mapping to mitochondrial genes were excluded from further analysis. Data were then normalized using SCTransform (22), scaled and the 3000 most variable features selected. For visualization, Harmony (0.1.1) was used to integrate cells from different individuals into a common embedding using the first 50 principal components (23). The first 30 harmony components were then used to create a t-distributed stochastic neighbor embedding using FIt-SNE (1.2.1) which was initialized using principal components (24).

Genes were considered specifically expressed when they classified a cell type (*versus* all other cells of any type) with an area under the receiver operating curve >0.8, or for testing the enrichment of cell types upon the partial least squares-discriminant analysis components, if their z-scored mean expression was >3.

#### Annotation of Cell Types

To annotate cell types gene expression profiles were integrated with the composite human heart reference dataset provided by Azimuth ((25, 26, 27), dataset: (28)). Cells were annotated by L1 cell type.

### Histology

#### Picrosirius Red Staining

Deparaffinized slides were transferred to Weigert’s hematoxylin (Sigma-Aldrich) for 10 min, then rinsed for 5 min in running tap water before transferring to picrosirius red solution (Sigma-Aldrich) for 15 min.

#### Tryptase Immunohistochemistry

Immunohistochemistry on 5 μm sections from human heart formalin-fixed paraffin-embedded (FFPE) samples was performed with the Roche Ventana DISCOVERY ULTRA machine following Protocol HQ. Monoclonal antibody 10D11 (Cell Signaling Technology, cat. no. 65164), reactive with mast cell tryptase, was used. Slides were baked at 60 °C for 32 min and deparaffinized at 72 °C for 24 min. Cell conditioning was conducted with CC1 at 95 °C for 40 min. DISC inhibitor was applied for horseradish peroxidase (HRP) blocking for 12 min. The primary antibody was used at 1:50 and was incubated at 37 °C for 60 min. Detection involved anti-rabbit HQ for 16 min followed by anti-HQ HRP for 16 min. 3,3ʹ-Diaminobenzidine (DAB) was used as the chromogen. Hematoxylin II was applied for 8 min, followed by Bluing Reagent for 4 min.

### Spatial Transcriptomics

Sections of 5-μm thickness were cut from FFPE blocks of human heart samples and mounted on Visium slides and processed for spatial transcriptomics according to the 10x Genomics Visium FFPE version 1 protocol. In brief, samples were deparaffinized, stained with H&E and imaged using a VS200 slide scanner (Olympus Life Science) before decrosslinking, destaining, and overnight probe hybridization with the 10x Visium Human version 1 probe set. The next day, hybridized probes were released from the tissue and ligated to spatially barcoded oligonucleotides on the Visium gene expression slide. Barcoded ligation products were then amplified and used for construction of libraries that were subsequently sequenced on a NovaSeq 6000 sequencing platform (Illumina), using a NovaSeq 6000 S2 Reagent Kit version 1.5 (Illumina) according to the manufacturer’s instructions.

#### Analysis of Sequencing Data

With Space Ranger version 2.0.0 (10x Genomics), reads were aligned to their corresponding probe sequences (Visium Human Transcriptome Probe Set version 1, based on GRCh38 2020-A) and mapped back to the Visium spot where a given probe was originally captured and, finally, aligned to the original H&E-stained image of the tissue section. The filtered count matrix.h5 file was used for further downstream processing and analysis of data.

Seurat 4.3 (21) was used for downstream analyses. Spots with data pertaining to <500 or >50,000 transcripts or <300 genes were excluded from further analysis. SCTransform (22) was used to normalize data within each slide, and then models were merged using the standard workflow. The 300 most variable features were scaled and for visualization Harmony (0.1.1) was used to integrate across different individuals into a common embedding using the first 50 principal components (23). A t-distributed stochastic neighbor embedding using FIt-SNE (1.2.1) was initialized using principal components (24). Spots were clustered using Louvain clustering, upon the kNN graph (k = 20). The resolution (tested in range 0.2–1.5 in intervals of 0.1) was selected which maximized the silhouette width calculated by Euclidean distance in PCA space.

Cell types were deconvolved using cell2location (29). A single reference cell type mean expression was calculated by negative binomial regression using all snRNAseq samples with the donor as a covariate, using genes common with the spatial transcriptomics dataset that were additionally detected in at least 3% of nuclei and with a mean nonzero expression of >1.12. Deconvolution was performed using hierarchical Bayesian models using the slide as the batch, with the sequencing batch as an additional covariate and with a prior of 8 cells per spot. We took the 5th percentile of the posterior distribution as the estimated cell type abundance. These values were subsequently Winsorised at 97.5th percentile.

mistyR (1.11.0) was used to predict the abundance of each cell type at each spot (30). The “intra” view contained the abundance of other cell types within a spot, the “juxta” view as the sum of the neighboring spots (within five edges in a graph constructed by 2D Delaunay triangulation) and the “para” view as spots within an effective radius of 15 spots and not present in the intra or para views. Estimation was performed separately on each slide and the mean variable importance or gain in R^2^ calculated.

### Cell Culture

#### Human Induced Pluripotent Stem Cell-Derived Cardiomyocytes

Human induced pluripotent stem-cell derived cardiomyocytes (5e6, iCell2 cardiomyocytes, donor 01434, from FujiFilm Cellular Dynamics) were thawed into RPMI containing B27 (plus insulin) supplement, 215 μg/ml ascorbic acid 2 phosphate sesquimagnesium (Sigma-Aldrich), and 1X penicillin streptomycin (Gibco). For the first 24 h after cell seeding, iMatrix 511 (1.8 μg/ml, AMSBio) was included to facilitate cell attachment and Y-27632 dihydrochloride (10 μM, Merck) was included to improve cell survival. After 4 days of post thaw recovery, cells were reseeded at a concentration of 20e^3^ per well in a PhenoVue 96-well plate (Revvity) and treated 2 days after with 10 nM chymase (Merck) and/or 50 μM chymostatin (Merck) in the cell culture medium. Plates were harvested for imaging and RNA 24 h after treatment.

Plates for imaging were washed twice with Hank's balanced salt solution (HBSS) (including calcium and magnesium, Gibco), stained for 15 min at 37 °C with CellMask Green Plasma Membrane stain (1:1000, Thermo Fisher Scientific), Cytotox Red (Sartorius) and Hoechst (1:1000, Thermo Fisher Scientific), then washed once more with Hank's balanced salt solution prior to fixation with 4% paraformaldehyde (Thermo Fisher Scientific) for 1 h at RT. Plates were imaged on an Operetta CLS high content imager, with 36 FOVs obtained per well with a 40X water immersion objective. Morphological analysis was conducted with Harmony 5.2.

Plates for RNA were washed twice with ice-cold PBS (no calcium or magnesium, Thermo Fisher Scientific), snap frozen on dry ice, and stored at −80 °C prior to RNA isolation, which was performed using the RNAdvance Cell v2 kit (Beckman Coulter) on a Biomek i7 liquid handling robot (Beckman Coulter) per manufacturer protocol. cDNA was synthesized using iScript reverse transcription supermix (Bio-Rad) per manufacturer protocol, prior to quantitative PCR (qPCR) using the probes described in Table 1 and TaqMan Fast Advanced Master Mix (Thermo Fisher Scientific) on a QuantStudio 12K Flex Real-Time PCR system (Applied Biosystems). Ct values were normalized to the housekeeping gene (RPLP0) included in each reaction in multiplex using the 2ˆ(-ΔCT) method (31).

**Table 1:** qPCR probe list

Gene	Supplier	Fluorophore	Catalog
RPLP0	Thermo Fisher	VIC	Hs99999902_m1
MYH6	Thermo Fisher	FAM	Hs01101425_m1
MYH7	Thermo Fisher	FAM	Hs01110632_m1
NPPA	Thermo Fisher	FAM	Hs00383230_g1
NPPB	Thermo Fisher	FAM	Hs00173590_m1
ACTA1	Thermo Fisher	FAM	Hs00559403_m1

qPCR, quantitative PCR.

### MAGnet RNAseq Analysis

Raw count data were obtained from the GEO (accession GSE141910) and analyzed using edgeR (32). Lowly expressed genes were removed by experimental group, and normalization was performed using the trimmed mean of M-values. Correlating principal components with metadata identified that most of the variance in the dataset overall (*i*.*e*.*,* component 1) related to RNA quality, and therefore outlying samples with a low RNA quality (median transcript integrity number <50) were removed. Dispersion was estimated tag-wise, and differential expression performed using a quasi-likelihood F-test (glmQLFFit and glmQLFTest). To adjust for batch effects, the sequencing batch was included as a covariate in the design matrix alongside disease status (PPCM, NPCM, and nonfailing control).

### Experimental Design and Statistical Rationale

PPCM is a rare disease, and we were only able to obtain myocardial tissue from patients that underwent heart transplant and for whom tissue had been collected from explanted hearts. In this exploratory study, we therefore utilized samples from all tissue donors with PPCM (n = 5, biological replicates) that were available within the Cardiovascular Biorepository at the University of Kentucky, and matched NPCM (n = 4, biological replicates) and control (n = 6, biological replicates) groups based upon age, body mass index and HbA1c status (Table 2). No technical replicates were analyzed. Since we anticipated that there would be many proteins for which we would be underpowered to detect per protein significant changes with this sample size, for analysis of tissue proteomes we utilized partial least squares discriminant analysis as a multivariate method to characterize the proteins that were most different between experimental groups, as implemented in the mixOmics R package (33).

**Table 2:** Clinical data of the patient cohort

Characteristic	CTRL, N = 6^a^	NPCM, N = 4^a^	PPCM, N = 5^a^	*p*-value
Age	43 (38, 55)	29 (27, 34)	31 (23, 37)	0.016∗^b^
Sex (Female)	6 (100%)	4 (100%)	5 (100%)	>0.9^c^
HbA1c (%)	5.40 (5.40, 5.40)	5.25 (5.07, 5.58)	4.90 (4.90, 4.90)	0.076^b^
Unknown	1	0	3	
Body mass index	26.9 (25.0, 30.6)	30.1 (27.0, 33.5)	28.1 (24.4, 30.5)	0.6*2*
Ejection fraction (%)	55 (55, 55)	22 (18, 26)	20 (20, 22)	0.010∗^b^
Unknown	1	0	0	
LV internal diameter diastole (cm)	4.40 (4.20, 4.40)	6.95 (6.70, 7.15)	6.60 (6.20, 6.70)	0.008∗∗^b^
Unknown	1	0	0	
LV internal diameter systole (cm)	3.10 (3.00, 3.30)	6.15 (5.65, 6.25)	5.60 (5.30, 5.80)	0.010∗^b^
Unknown	1	0	0	
Prescribed medication for heart failure	0 (0%)	4 (100%)	5 (100%)	<0.001∗∗∗^c^

Left ventricular myocardial tissue samples were obtained from six female organ donors without a history of cardiovascular disease (CTRL) as well as five PPCM patients and 4 NPCM patients who received a heart transplant or left ventricular assist device due to end-stage disease. Ejection fractions were decreased in NPCM and PPCM patients compared to controls, and left ventricular diameters at both end-diastole and end-systole were increased in cardiomyopathy patients regardless of etiology. Angiotensin converting enzyme (ACE) inhibitors, angiotensin receptor blockers (ARB), aldosterone antagonists, or beta blockers were considered as prescribed medication for heart failure, unless an alternative reason for their prescription was recorded in the medical history. ∗∗∗*p* < 0.001, ∗∗*p* < 0.01, ∗*p* < 0.05.

a Median (IQR); n (%).

b Kruskal–Wallis rank sum test.

c Fisher's exact test.

### Statistical Analysis

Data are presented as the mean ± standard deviation, unless otherwise stated. Unless otherwise stated data analysis was performed using R (34). *p* < 0.05 was considered significant, or where multiple tests are being performed a FDR less than 0.05, as calculated by the method of Benjamini and Hochberg (35). Gene set enrichment was calculated after the method by Subramanian et al. (36) using fgsea (37). Overrepresented gene sets were calculated using the hypergeometric test (38). Logistic regression was performed on scaled (mean = 0, standard deviation = 1) aptamer abundance in order to facilitate interpretation. The *F*-test unless otherwise stated, assumed equal variance between groups.

## Results

### Clinical Cohort

We obtained left ventricular myocardial samples from six organ donors, as well as five patients with PPCM and four NPCM patients receiving either a heart transplantation or implantation of a left ventricular assist device. As presented in Table 2, all patients were female and groups were matched for age, body mass index, and HbA1c status. Both PPCM and NPCM patients had reduced left ventricular ejection fraction and increased left ventricular diameter at both end-diastole and end-systole compared to controls. No organ donor had been prescribed medication for heart failure.

### Both PPCM and NPCM Hearts Show Proteomic Signatures of End-Stage Heart Failure

**Fig. 1:**
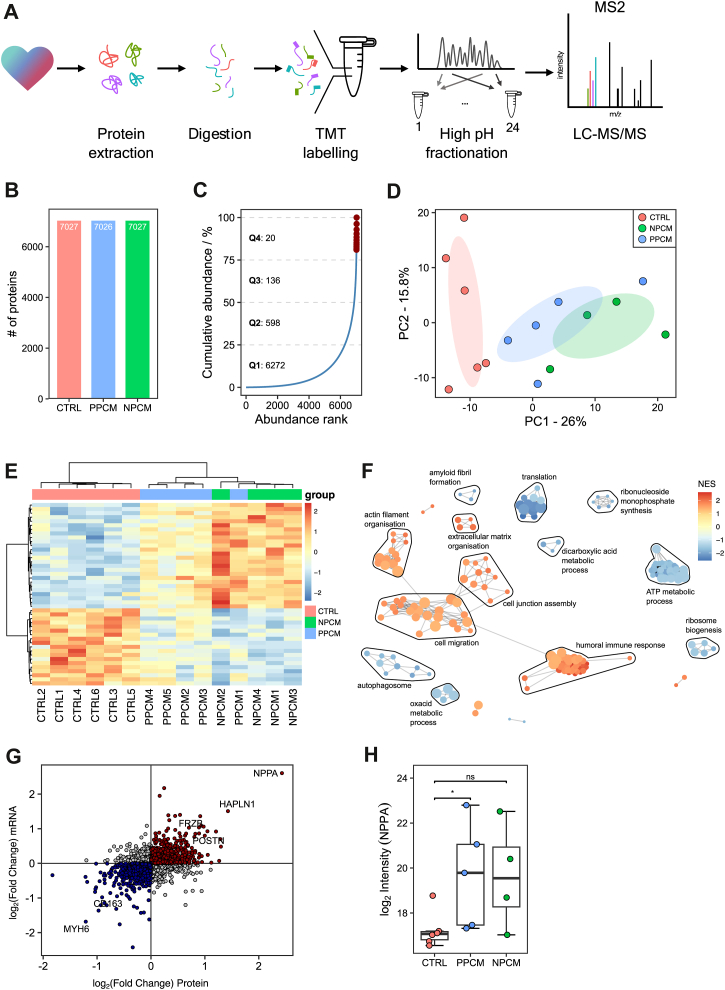
**Proteomic profile of PPCM, NPCM, and control hearts**. *A*, schematic overview of quantitative proteomics workflow applied to human heart tissue for global protein profiling. Proteins were quantified by reporter ion intensities of TMT-labeled peptides. *B*, analytical depth of quantitative proteomics approach. Number of proteins quantified with at least two peptide spectrum matches per protein and without missing value imputation. *C*, cumulative protein abundance in our dataset of human heart tissue as a function of abundance rank. Number of proteins contributing to each quantile is indicated and Top 10 most abundant proteins are highlighted. *D*, principal component analysis (PCA) of protein abundances of PPCM, NPCM, and donor hearts shows a clear separation of diseased hearts from donor hearts. *Shaded regions* depict the 95% confidence level for a multivariate *t*-distribution. *E*, heatmap of 45 proteins that are differentially abundant between groups, at a false discovery rate of 0.05. *F*, network plot of Gene Ontology biological processes that are significantly enriched between heart failure of any etiology and control (upregulated in *red*, downregulated in *blue*, nodes represent individual enriched gene sets that are clustered based upon their similarity, with singletons not shown). *G*, correlation of the fold change between heart failure (of any etiology) and control in the proteomics of this study, and a meta-analysis of transcriptomics studies (41) shows a significant correlation that demonstrates the external validity of our cohort. *Red* = concordant positive fold change, Blue = concordant negative fold change. *H*, Log_2_-transformed NPPA intensities in CTRL, PPCM, and NPCM samples. *Boxes*: interquartile range (IQR); lines: median; whiskers: min/max within 1.5 × IQR. Significance (Mann–Whitney U test) is shown as ∗ (*p* < 0.05) and ns (not significant), with PPCM *versus* CTRL *p* = 0.017, NPCM *versus* CTRL *p* = 0.11. HF, heart failure; LV, left ventricle; LC- MSMS, liquid chromatography coupled to tandem mass spectrometry; NPCM, nonperipartum cardiomyopathy; PC1, principal component 1; PC2, principal component 2; PPCM, peripartum cardiomyopathy; TMT, tandem mass tag.

To investigate differences in the molecular profile of PPCM hearts compared to NPCM hearts and hearts of organ donors, we applied an unbiased deep quantitative proteomics approach. Proteins were extracted from myocardial tissue, digested into peptides and labeled with chromatographically indistinguishable isobaric TMT, which allow multiplexing of samples and result in precise relative quantification of protein abundance between samples. To increase coverage of the proteome, samples were extensively fractionated offline to reduce their complexity before subsequent analysis by LC-MS/MS (Fig. 1*A*). Subsequently, 7026 proteins were quantified by 172,946 peptide-spectrum matches across all 15 heart samples thus achieving a deep proteomic dataset for comparison across hearts in PPCM, NPCM, and nonfailing controls (Fig. 1*B*, Supplemental Fig. S1, *A*–*E*).

The proteomic profile of human heart tissue has an exceptionally high dynamic range (39, 40), which poses a challenge for covering lowly abundant proteins in the sample. This high dynamic range of the cardiac proteome was reflected in our dataset as on average only 156 proteins contribute to 50% of the total protein abundance (Fig. 1*C*). Among the 10 most abundant proteins were constituents of the myofibril (MYH7, TTN, MYL3, MYL2, MYBPC3, and TPM1), mitochondria (ATP5F1, ACO2, and CKM) and myoglobin (MB). Despite this analytical challenge, we successfully covered proteins across a dynamic range spanning more than 5 orders of magnitude, enabling a deep quantitative evaluation.

Principal component analysis of all measured protein abundances demonstrated a clear separation of heart samples from patients with cardiomyopathy from those of non-failing controls along component 1, which suggested that the primary source of variance in our dataset is related to the disease phenotype (Fig. 1*D*) and not other clinical covariates (assessed by Pearson correlation between principal components and clinical covariates; Supplemental Fig. S2*A*). Many of the proteins which contributed the most to this separation of failing hearts from nonfailing hearts have been described before to be regulated in heart failure (MYH6, POSTN, HAPLN1, and SAA1), or are typical heart failure markers like atrial natriuretic peptide (ANP, NPPA; Supplemental Fig. S2*B*).

Testing for proteins whose abundance differs between any of the three patient groups identified 45 proteins that are differentially abundant (empirical bayes moderated *F*-test; all statistically significant proteins are visualized in Fig. 1*E*). Consistent with the principal component analysis, the majority of the difference was observed between cardiomyopathy of any etiology and controls (Supplemental Fig. S2*C*). Testing for enriched biological processes based upon changes in protein abundance between end-stage heart failure and control recapitulated known changes in heart failure, such as an upregulation of extracellular matrix organization and a downregulation of ATP metabolic processes and translation (Fig. 1*F*).

To validate our observations with external cohort data, we compared the differences in protein abundance we observed between cardiomyopathy and control with differences in mRNA expression derived from a comprehensive transcriptome “consensus signature” of heart failure of mixed etiology (41). This is a broader set of patients than those with only dilated cardiomyopathy utilized here. Of these, 6475 gene products were common between our proteomics and the published transcriptomics datasets. There was a good correlation in measured fold changes of gene products between heart failure patients and controls (rho = 0.42; Fig. 1*G*) showing that the direction of change observed in this cohort at the protein level is consistent with the direction of change observed at the transcriptional level of the larger cohort. The most changed gene product between heart failure and control across both cohorts was atrial natriuretic peptide (NPPA; Student’s *t* test, *p* = 0.011; protein abundance in this study shown in Fig. 1*H*). We therefore concluded that the molecular profile of our cohort is representative of changes that occur with end-stage cardiomyopathy in the wider patient population.

### Proteome Remodeling in PPCM Hearts Aligns With Transcriptional Differences Observed in an Independent Cohort

**Fig. 2:**
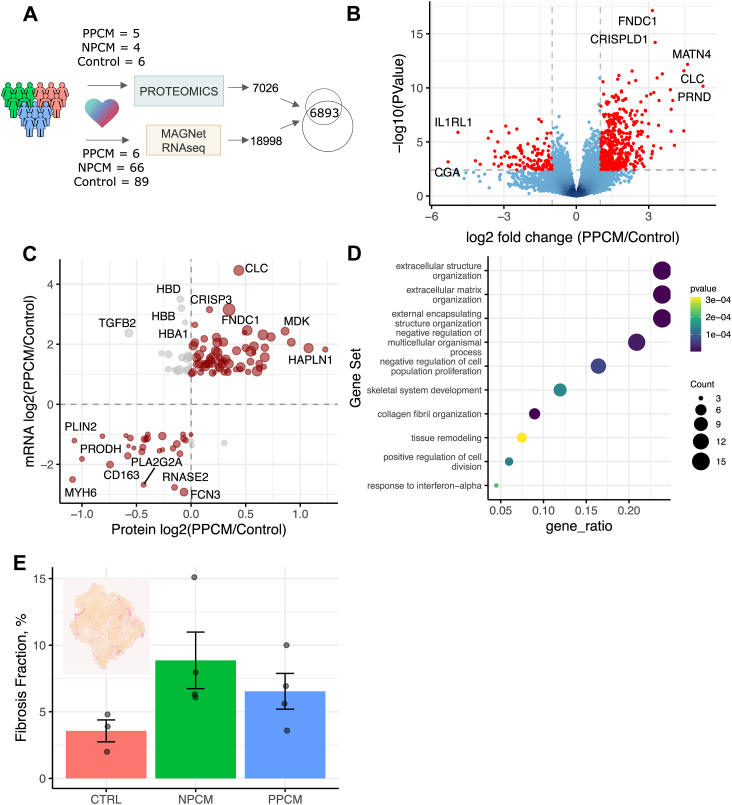
**Concordant changes with an external cohort measured at the level of transcripts shows external validity**. *A*, RNAseq data from the from the MAGnet consortium includes a number of PPCM samples, providing the opportunity to intersect the changes observed 6893 gene products quantified both at the level of proteins in this study with the transcripts measured in an external cohort. *B*, reanalysis of the publicly available RNAseq dataset from the MAGNet consortium identifies 588 transcripts that are differentially regulated between hearts with end-stage PPCM in comparison to control. *C*, intersection of fold changes between PPCM hearts and control hearts quantified at the level of protein abundance (this study) with fold changes of mRNA of the significantly differentially expressed genes (MAGNet consortium dataset) shows the external validity of our cohort through concordant changes. *D*, gene sets of Gene Ontology biological processes overrepresented in those gene products that are statistically upregulated in PPCM compared to control in the MAGnet transcriptome, and have a positive fold change in the tissue proteome. The 10 gene sets with the smallest *p* value are shown. *E*, picrosirius red staining was used to quantify the fibrosis fraction of tissue samples, showing an increase in both heart failure groups in comparison to control (ANOVA, *p* = 0.0007). Inset shows an exemplar slide from the PPCM group. PPCM, peripartum cardiomyopathy.

As PPCM is a rare disease, we were faced with the limitation of studying a small cohort. To compensate for this limitation and address the external validity of the remodeling that occurs specifically in PPCM, we sought to compare the results of our proteomics investigation with a complimentary dataset collected by the MAGNet consortium (GEO accession GSE141910). Similar to our own cohort, the MAGNet consortium also included hearts of six PPCM patients, in addition to a large number of NPCM (n = 66) and organ donor control hearts (n = 89; Fig. 2*A*). They then measured the transcriptome by RNA sequencing. Analyzing these data while accounting for batch effects, we found that 588 transcripts differed significantly between PPCM patients and controls (Fig. 2*B*). Of these 588 transcripts that are significantly regulated in PPCM hearts, we saw a consistent direction of regulation at the level of protein abundance in the PPCM cohort we studied (Fig. 2*C*), although the proteins do not reach statistical significance (Supplemental Fig. S2*D*). Among the consistently upregulated gene products were PI16, proposed as a fibroblast marker across tissues (42); AEBP1, which has recently been identified as essential for fibroblast activation (43); and the extracellular matrix proteins HAPLN1 and MFAP4. Consistent with these observations, proteins with concordant regulation across both cohorts and modalities and whose abundance increased in PPCM were enriched for biological processes related to extracellular matrix organization and the immune response (Fig. 2*D*; decreased abundance shown in Supplemental Fig. S2*E*). To confirm that extracellular matrix remodeling brought about an increase in fibrosis, we stained tissue sections with picrosirius red which confirmed an increase in the density of collagen in hearts from both NPCM and PPCM groups compared to controls (*F*-test, *p* = 0.0007; Fig. 2*E*). Our proteomics data for PPCM hearts are therefore also supported by congruent observations in an external PPCM cohort.

### Remodeling of Mast Cell Proteins CMA1 and CPA3 is Specific to PPCM Hearts

**Fig. 3:**
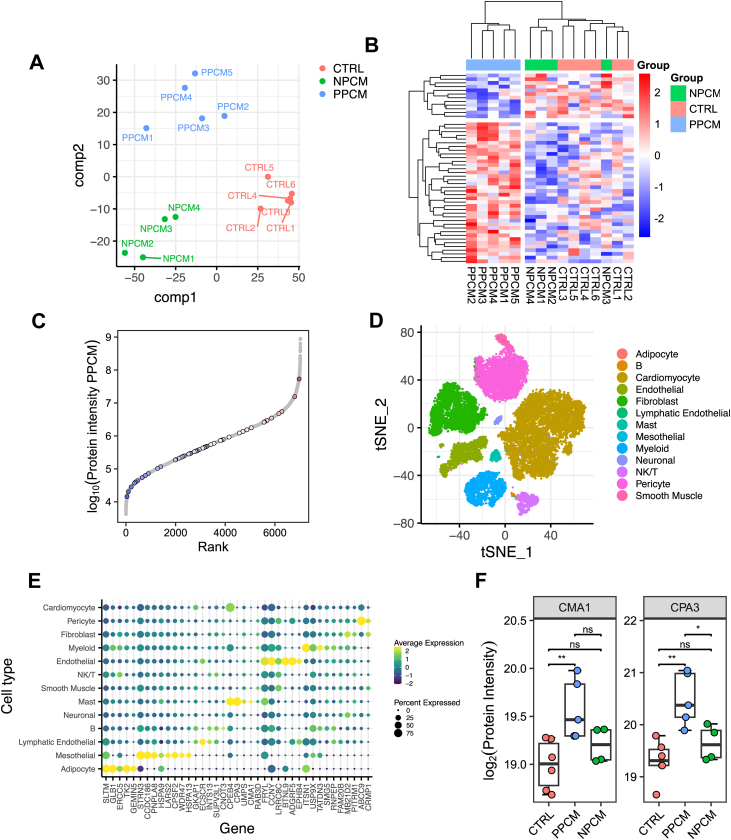
**Discriminant analysis identifies proteins whose expression is altered specifically in PPCM**. *A*, partial least squares discriminant analysis separates all three experimental groups. Component 1 separates heart failure (of any etiology) from control samples, whereas component 2 separates PPCM specifically from both other groups *B*, protein-wise z-scored abundance of the 50 proteins contributing most to the separation of PPCM along component 2. *C*, the abundance of the 50 proteins contributing most to component 2 in the context of the entire dataset. *D*, t-distributed stochastic neighbor embedding showing the cell type of 22,049 nuclei derived from the same tissue samples as used for the proteomics. *E*, intersection of tissue proteomics with cell type gene expression profiles from single nucleus RNAseq identifies the likely cell type of origin for the same 50 proteins. *F*, log_2_-transformed protein abundances of CMA1 and CPA3, which are predominantly expressed in mast cells and among the proteins most positively associated with PPCM. *Boxes*: interquartile range (IQR); *lines*: median; whiskers: min/max within 1.5 × IQR. Significance (Mann–Whitney U test) is shown above comparisons as ∗∗ (*p* < 0.01), ∗ (*p* < 0.05) or ns (not significant). CMA1: PPCM *versus* CTRL ∗∗ (*p* = 0.0043), PPCM *versus* NPCM ns (*p* = 0.19), NPCM *versus* CTRL ns (*p* = 0.26). CPA3: PPCM *versus* CTRL ∗∗ (*p* = 0.0043), PPCM *versus* NPCM ∗ (*p* = 0.032), NPCM *versus* CTRL ns (*p* = 0.26). NPCM, nonperipartum cardiomyopathy; PPCM, peripartum cardiomyopathy.

To address if there is any protein remodeling that can distinguish PPCM from the largely similar NPCM hearts, we employed discriminant analysis in order to identify (combinations of) proteins whose abundance most strongly differentiates the three patient groups in our cohort. This showed that patient groups could be successfully distinguished, including resolving the differences between PPCM and NPCM (Fig. 3*A*). Here, component 1 captured differences between cardiomyopathy and control, while component 2 separated specifically PPCM from both other groups. The relative abundances of the 50 proteins that contributed most to the separation specifically of PPCM are shown in Figure 3*B* and their loadings upon component 2 in Supplemental Figure S5*A*. Many of these proteins are found to have a low median abundance (Fig. 3*C*), highlighting the importance of our extensive sample fractionation in order to measure not only highly abundant proteins.

We sought to understand the cellular context of the protein signature particular for PPCM hearts. To this end, we performed single nucleus transcriptomics (snRNAseq) analysis of the same heart samples that were analyzed by proteomics (Supplemental Fig. S3, *A*–*G*). After rigorous quality control we measured 22,049 nuclei isolated from the human heart samples (biological replicates: PPCM n = 4, NPCM n = 4, control n = 4; Supplemental Fig. S4, *A*–*C*). The resulting data are shown labeled according to their cell type in the nearest neighbor embedding of Figure 3*D*. We utilized this dataset to address which cardiac cell types express the proteins that we identified to have a particular profile in PPCM hearts (protein identities shown in Supplemental Fig. S5*A*). Figure 3*E* shows the transcript expression profiles across cardiac cell types for these proteins. We turned our attention to immune cell populations due to the enrichment in immune related processes among the concordant changes seen with the transcriptome of the external PPCM cohort. Among the proteins where the snRNAseq data pointed toward cell type specific expression were an increase in the abundance of the mast cell proteases CMA1 (chymase; protein abundance shown in Fig. 3*F*) and CPA3 (carboxypeptidase A3; protein abundance shown in Figure 3*F*, cell type resolved gene expression shown in Supplemental Fig. S4*F*). Both proteins are specifically more abundant in the PPCM hearts.

To determine whether increased CMA1 and CPA3 abundance was due to a change in mast cell phenotype or to an increase in the number of mast cells within the tissue, we measured the number of mast cells by immunohistochemical staining against tryptase, a mast cell marker not quantified in our proteomics dataset (Supplemental Fig. S5*B*). As in prior studies (44) mast cells were rare in experimental samples. We did not observe any statistical differences in their areal density across the three patient groups (ANOVA without assuming equal variance, *p* = 0.1103; Supplemental Fig. S5, *B*–*C*). Concordant with observing no change in the quantity of mast cells, there is no overall enrichment of mast cell specific proteins defined using atlas single-cell gene expression datasets (45) among the components associated with HF of any etiology or those with PPCM in our discriminant analysis (Supplemental Fig. S5, *E*–*F*). These two orthogonal pieces of evidence both suggest that the increased abundance of CMA1 and CPA3 specifically in PPCM is due to a change in mast cell phenotype rather than quantity.

### Mast Cells Are Located in the Immediate Vicinity of Fibroblasts

**Fig. 4:**
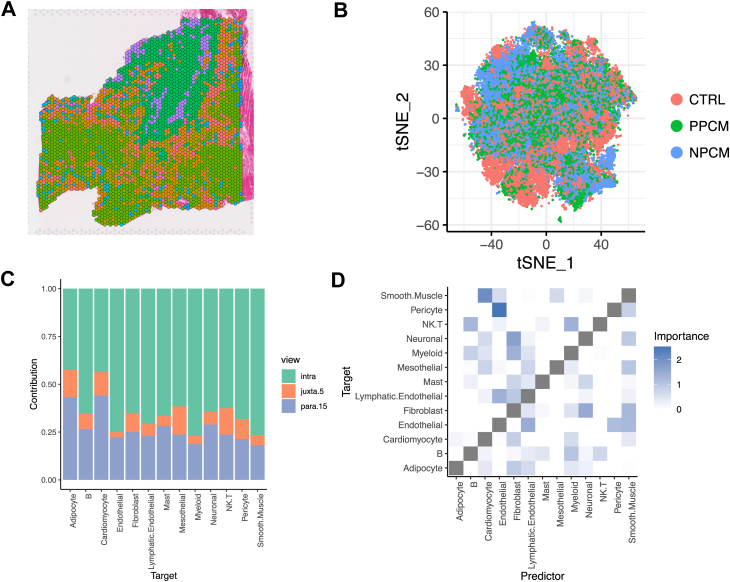
**Mast cells are found located in the immediate vicinity of fibroblasts and promote a fibrotic phenotype**. *A*, exemplar slide, with spots clustered based upon gene expression. *B*, neighbor embedding, showing all spots across all samples included in the spatial transcriptomics experiment, again clustered by gene expression. *C*, spatial transcriptomics data were deconvolved to calculate the abundance of all cell types at each spot in the tissue. Abundance of each cell type at each spot was predicted using the abundance of other cell types at that spot in the tissue, in the surrounding neighborhood and from further away in the tissue (stylized in inset). The performance of the model in predicting mast cell abundance is mostly due to the contribution of the abundance of cell types that are located in the immediate vicinity. *D*, in predicting mast cell abundance using information from the immediate vicinity, fibroblasts are the most important cell type.

Hypothesizing that mast cells interact with other cell types in their immediate vicinity, we performed spatial transcriptomics in order to characterize how such a change in mast cell phenotype impacts the surrounding tissue. After quality control, we measured the gene expression at a mean of 2911 locations (diameter 55 μm) across biological replicates (PPCM n = 4, NPCM n = 4, control n = 5; Fig. 4, *A*–*B*, Supplemental Fig. S6, *A*–*B*). Using cell type reference profiles derived from our single nucleus transcriptomics data, we deconvolved the expression profile at each spot to calculate an estimated abundance of each cell type present in our single nucleus transcriptomics data (Supplemental Fig. S6*C*). The average estimated cell type composition across each sample correlated well with that calculated directly from the single nucleus transcriptomics (rho = 0.80; Supplemental Fig. S6*D*). We then explored how interactions between cells in (a) the immediate environment, (b) the local neighborhood and (c) the broader tissue structure predicted abundances of each cell type, using a previously described machine learning model (30). Most predictive information was found in the immediate environment, including for mast cells (Figs. 4*C*, Supplemental Fig. S6*E*). The abundance of both fibroblasts and neuronal cells in the immediate vicinity were both predictors of mast cells (Fig. 4*D*). These results suggest that mast cells are localized in immediate vicinity of fibroblasts. This may suggest a link between the fibrotic phenotype observed in PPCM and increased abundance of the mast cell proteases CMA1 and CPA3.

### Chymase Negatively Impacts Cardiomyocyte Phenotype

**Fig. 5:**
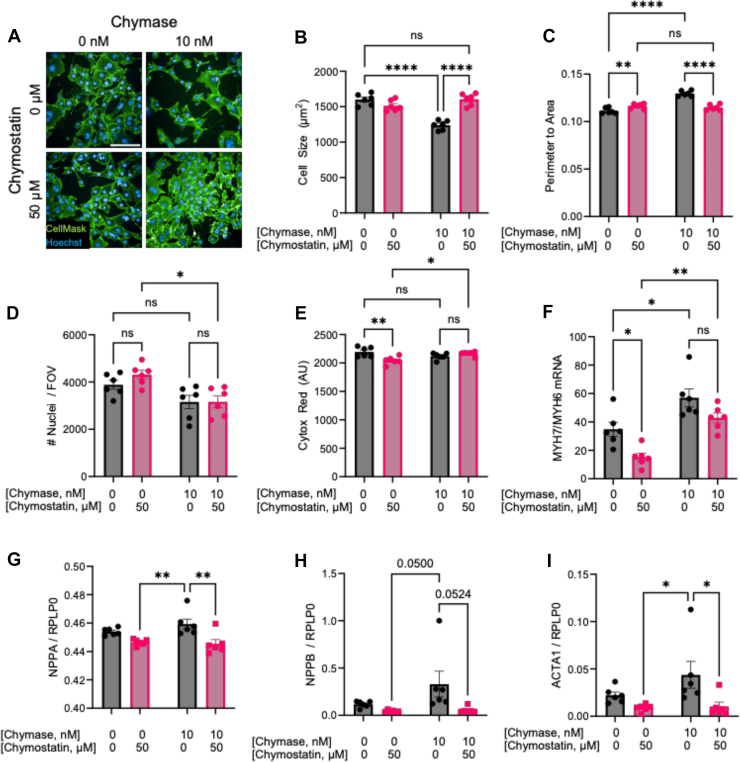
**Chymase affects human cardiomyocytes *in vitro***. *A*, representative images of human induced pluripotent stem cell-derived cardiomyocytes (hiPSC-CMs) treated for 24 h with 10 nM chymase with and without 50 μM chymostatin and stained with CellMask (plasma membrane) and Hoechst (nuclei). *B*, chymase treatment caused a decrease in cell area that was prevented by inhibition with chymostatin. *C*, chymase treatment caused an increase in the perimeter to area ratio (meaning that cells are becoming less round), that was prevented by inhibition with chymostatin. *D*, there was no change in cardiomyocyte number with chymase treatment. *E*, there was no measurable cytotoxicity with chymase treatment. *F*, the ratio of *MYH7* to *MYH6* measured by qPCR specifically increased following treatment with chymase. *G*, *NPPA* expression showed a trend toward increase with chymase treatment, with a significant decrease upon chymostatin inhibition. *H*, *NPPB* expression showed a trend toward increase with chymase treatment, with a significant decrease upon chymostatin inhibition. *I*, chymase treatment significantly increased the expression of *ACTA1*, which was prevented by the presence of chymostatin. ∗∗∗∗*p* < 0.0001, ∗∗∗*p < 0*.*001*, ∗∗*p < 0*.*01*, ∗*p* < 0.05 by two-way ANOVA with Tukey’s post hoc test. qPCR, quantitative PCR.

As a form of systolic heart failure, PPCM is characterized by decreased contractile function. We therefore sought to evaluate if increased chymase might impact cardiomyocytes directly. Human induced pluripotent stem-cell derived cardiomyocytes were treated *in vitro* for 24 h with and without chymase and its inhibitor chymostatin and membranes stained to enable quantification by high content imaging. Chymase specifically elicited a change in cardiomyocyte morphology (exemplar images shown in Fig. 5*A*), which resulted in smaller cardiomyocytes with more protrusions, quantified by a decrease in cell area and increase in perimeter to cell area ratio (Fig. 5, *B*–*C*). There was no change in cardiomyocyte number nor measurable cytotoxicity with chymase treatment (Fig. 5, *D*–*E*). A transcriptional shift to myofilament gene expression was also observed by quantitative PCR, marked by an increase in the ratio of *MYH7* to *MYH6* (Fig. 5*F*) indicative of a cardiomyocyte stress response. Consistent with the myofilament isoform changes, we observed trends toward increased transcription of *NPPA* and *NPPB*, encoding the A and B-natriuretic peptide proteins, and a significant decrease when chymase was inhibited using chymostatin (Fig. 5, *G*–*H*). The gene encoding skeletal muscle actin (*ACTA1*), which is fetally expressed and reactivates in failing cardiomyocytes in NPCM followed a similar pattern, with a significant decrease upon chymostatin treatment (Fig. 5*I*). Increases in chymase are therefore sufficient to negatively impact the cardiomyocyte phenotype, and thus chymase could be mechanistically relevant to disease progression.

### Chymase as a Diagnostic Marker of PPCM in Blood Serum

**Fig. 6:**
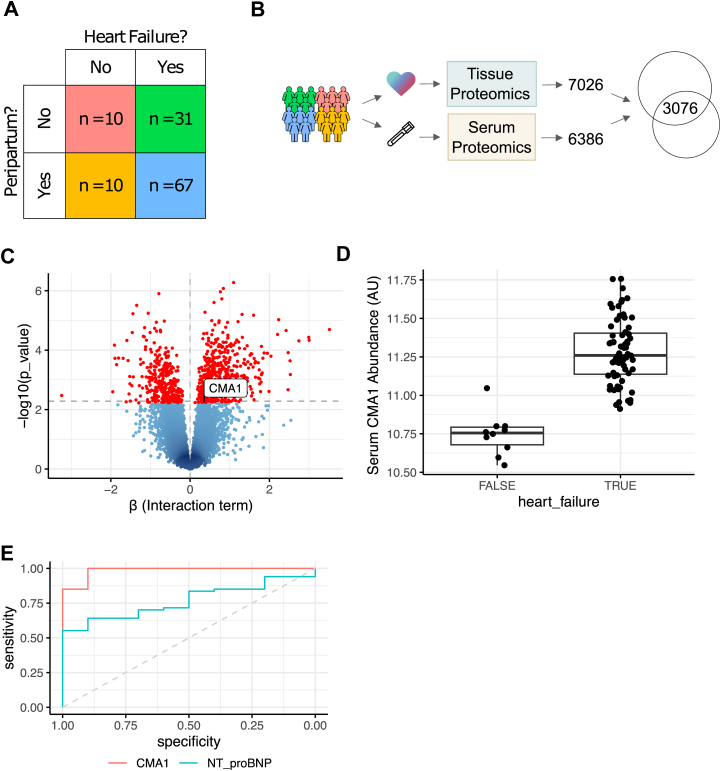
**CMA1 is increased in peripheral blood serum collected shortly after diagnosis of PPCM**. *A*, when measuring the proteome of the blood serum a full factorial experimental design was used: across all possible combinations of individuals both with and without peripartum status and with and without cardiomyopathy. PPCM patients for example are recently postpartum and have cardiomyopathy. This is in contrast to the tissue proteome, where we were unable to obtain tissues from peripartum women without cardiomyopathy (*i*.*e*.*,* PPHC). *B*, A total of 3076 proteins were quantified both in heart tissue in this study, and in peripheral blood serum using the SomaScan assay by Lovell *et al* (46). *C*, One hundred ninety-four uniquely identified proteins have a significantly higher or lower expression in PPCM than would be observed based upon the additive effect of pregnancy and heart failure, indicating that they are differentially regulated specifically in PPCM. *D*, abundance of CMA1 in peripheral blood serum is increased in PPCM in comparison to NPCM (Welch’s *t* test, *p* < 0.0001). *E*, logistic regression was used to predict the probability of heart failure from CMA1 abundance. The receiver operating characteristic shows that CMA1 has excellent discriminative performance in distinguishing PPCM from pregnant controls without heart failure (area under receiver operating characteristic for CMA1 = 0.985, for N-terminal proBNP = 0.767). NPCM, nonperipartum cardiomyopathy; PPCM, peripartum cardiomyopathy.

A major shortcoming of investigations of PPCM is the inability to study heart tissue samples from women who are pregnant but without heart disease, leaving pregnancy as a confounding factor in the experimental design. We sought to mitigate against the confounding impact of pregnancy on our conclusions by incorporating a recently published serum proteome dataset (46). This was derived from peripheral blood samples collected shortly after parturition from women with PPCM, NPCM, nonperipartum healthy controls and crucially also peripartum healthy controls (PPHC; Fig. 6*A*). In addition, 3076 proteins were common with those quantified in our analysis of the tissue proteome (Fig. 6*B*) and for these we tested for an interaction between peripartum status and the presence of heart failure. A significant interaction term (either positive or negative) would imply that protein abundance is altered specifically by PPCM, rather than being attributable to the impact of either pregnancy or cardiomyopathy alone. One hundred ninety-four uniquely identified proteins were found to be specifically regulated by PPCM (empirical bayes moderated *t* statistic; Fig. 6, *C*–*D*). CPA3 was not measured in this aptamer-based proteomics panel, but notably CMA1 had a positive interaction between peripartum status and the presence of cardiomyopathy (*p* = 0.006, significant at a FDR of 0.0503; Fig. 6*C*). Increased CMA1 abundance thus cannot be attributed solely to the recent peripartum status of PPCM patients but is specific to the disease phenotype.

Increased CMA1 abundance in the myocardial tissue specifically of PPCM patients led to the hypothesis that CMA1 abundance might also be specifically increased in the peripheral blood of PPCM patients. We therefore tested if CMA1 in peripheral blood could be used to identify PPCM in peripartum women using logistic regression (PPCM n = 67, peripartum without PPCM n = 10). The results showed that chymase outperformed the currently utilized NT-proBNP (47). Chymase abundance was however not informative about cardiac recovery within the first 12 months following diagnosis (Fig. S7*B*) and unlike in cardiac tissue its abundance in the serum was not highly specific to PPCM compared to NPCM (Fig. S7*C*). In combination with the evidence that chymase is specifically upregulated in PPCM and is mechanistically relevant to cardiomyocyte phenotype, we therefore propose CMA1 as a candidate diagnostic biomarker for PPCM.

## Discussion

We applied unbiased methodologies using multiple data modalities to characterize how PPCM modulates molecular and cellular properties relative to NPCM and nonfailing hearts. Lending credence to our data, we recapitulate many known changes between end-stage heart failure (of any etiology) and nonfailing controls. Within heart tissue, we observe specific changes in PPCM in a subset of proteases expressed by mast cells within the immune compartment (notably CMA1 and CPA3) which are supported by evidence in external cohorts. CMA1 measured in the blood serum is able to predict PPCM diagnosis in pregnant women.

### Immune Activation in PPCM

An increase in CMA1 and CPA3 is consistent with a role of immune cell involvement in PPCM, which has long been supported by epidemiological data (48) and more recently with molecular insights (46). Current mechanistic hypotheses about the pathophysiology of PPCM center upon the role of processes downstream of the hormone prolactin (49). An immune component of the pathology is consistent with these hypotheses: outside the canonical action of prolactin in lactation in nursing mammals, perhaps its major role is in immune regulation (50).

### Mast Cells

CMA1 and CPA3 are both expressed specifically within mast cells. Mast cells are a rare cell type in the heart, with heterogenous functions that have led to them being labeled as a “contextual chameleon” (51). Their role in pathophysiology is relatively less well characterized compared to more abundant immune cell types (52, 53). It has been previously reported that mast cell density in 2D sections is increased in the failing heart, agnostic to the etiology (44, 54, 55). We note that the number of cells within a 2D section does not only depend on the number of cells within a tissue volume, but also upon the size of the cells (56) which methods identifying mast cells by their granule contents are unable to assess. Our data provide no evidence for an increase in mast cell density, either using analogous methods counting tryptase positive regions in 2D after immunohistochemistry, or in an -omics approach by measuring the abundance of all quantified proteins whose gene expression is specific to mast cells compared to other cell types.

Chymase is an endopeptidase that is found both within the interstitium of the heart and in granules within the cytoplasm of mast cells (57). CPA3 is on the other hand an exopeptidase, preferentially cleaving C-terminal aliphatic amino acids (58). They are commonly found not only stored together in the same granules but physically interacting (59), which is thought to reflect synergistic functions in protein cleavage, as the endopeptidase activity of chymase creates new substrates for subsequent exopeptidase activity by CPA3 (60). Among substrates for its endopeptidase activity, chymase has been shown to cleave angiotensin I to form angiotensin II (61, 62), whose canonical actions results in increased blood pressure. Indeed it is now known that >75% of the production of angiotensin II within the human heart is due to the action of chymase (61) rather than renin. This may be pathologically relevant since PPCM patients are enriched for hypertensive diseases of pregnancy, including preeclampsia (63). Preventing such increases in chymase activity could thus be a viable target in the treatment of PPCM.

Mast cells are furthermore known to play a central role in tissue remodeling in cardiovascular disease, which has led to existing attempts to alter their function, such as to prevent the pathological tissue remodeling after myocardial infarction (64). Most simply, mast cell function can be inhibited using a mast cell stabilizer that prevents degranulation. Chronic mast cell stabilization in the dog however results in a decrease in left ventricle ejection fraction, with corresponding decreases in contractile function also seen in isolated cardiomyocytes (64). This is patently not desirable in the setting of PPCM. Mast cells have also been shown to play a dual role in tissue remodeling (65, 66) and hence untargeted strategies to inhibit mast cells might abrogate benefits as well as mitigate detriments.

A more targeted alternative approach to inhibiting degranulation is to inhibit specific contents of mast cell granules directly. Increases in cardiac chymase activity have been reported in preclinical models of heart failure (67, 68); however this finding has not been not consistent, with no difference reported elsewhere (69, 70). Increased chymase activity was also observed in clinical heart failure samples (71). This led to a range of compounds have being developed to specifically inhibit chymase activity (72). The most advanced of these being developed for use in cardiovascular disease was Fulacimstat, a first-in-class molecule being tested for use to mitigate heart remodeling after myocardial infarction (72). However at phase 2, no evidence for increases in left ventricular ejection fraction as the primary endpoint was reported (73). If, in concordance with our data, PPCM were a more appropriate disease indication for either this molecule or other chymase inhibitors, safety in pregnant or breastfeeding women beyond that demonstrated in phase 1 would need to be ensured (73).

#### Do Mast Cells Play a Causative Role in the Pathology?

We show that mast cells are preferentially located in the vicinity of fibroblasts and neuronal cells. Colocation with neuronal cells has previously been described with histology of tissue surrounding coronary arteries (74). Mast cells within fibrotic regions have also been previously observed in human failing heart (75, 76), and positive correlations have been identified between the number of mast cells and the extent of myocardial fibrosis in cardiomyopathy (75, 76). It has also been shown that mast cells can stimulate procollagen I production from fibroblasts (77). This is consistent with the known role of mast cells in remodeling of heart tissue, which has been best studied following myocardial infarction (75, 76). Chymase is a canonical upstream regulator of matrix metalloproteinase activity (78, 79), and indeed a quantitative relationship has been observed between mast cell number and MMP activity in the heart (80). In model systems, coculture of mast cells and fibroblasts causes MMP9 release (81, 82). Fu et al. show that this is dependent upon the actions of chymase: stretch of fibroblasts is sufficient to induce chymase production within the fibroblast, and treatment with a chymase inhibitor reduces stretch-induced autophagy in isolated cardiac fibroblasts.

Intriguingly, an interaction between circulating estrogen levels and cardiac mast cell phenotype has been previously reported. By ovariectomizing rats and then subsequently exogenously restoring circulating estrogen levels, Chancey et al. showed that mast cells caused ventricular dilatation only when estrogen levels were below normal levels (83). This is consistent with other reports of a direct effect of estrogen upon other mast cell populations (84, 85, 86). One might speculate about a role in PPCM for a failure of the cardiac mast cells to adapt to the postpartum *milieu* as plasma estrogen levels fall after parturition.

#### Diagnostic Approaches in PPCM

Diagnosis of PPCM is often challenging, due to an overlap of symptoms of heart failure with not only normal characteristics of pregnancy (such as breathlessness and fatigue) but moreover symptoms of other complications of pregnancy such as preeclampsia (3). In our analysis of data from a proof-of-principle cohort CMA1 had excellent performance in discriminating PPCM patients from healthy controls in recently postpartum individuals. There are a variety of molecules that have previously been proposed as biomarkers for either diagnosis or prognosis of PPCM (87, 88), but recent position statements emphasize the need for further research before recommendations are made (47). This is the case equally for CMA1. The diagnostic performance of CMA1 needs be confirmed in a larger independent cohort, but also its specificity for PPCM in comparison to other complications of pregnancy needs to be determined. This is especially important, given reports of increased chymase activity in plasma of preeclamptic compared to normal pregnancies (89). We note that in such cases its diagnostic performance is independent from its actions within the heart, with for example the increase in plasma chymase during preeclampsia thought to originate from the placenta (90), and not from heart tissue.

### Limitations

The main limitation is the small sample size. However, PPCM is a rare disease complicating data collection. Although we confirm the external validity of our findings beyond our own cohort by integrating our own data with publicly available data from independent patient populations, we anticipate that there are many additional relevant changes which we did not have the statistical power to detect. This highlights the importance of collaborative initiatives, either at continental or global scales (91, 92) in order to continue to generate, test and validate hypotheses.

Our analytical approach prioritized the identification of high-confidence signals by focusing on convergent changes *i*.*e*.*,* those changes conserved between protein and transcript abundance. We recognize that discrepancies between these data modalities can also provide valuable biological information, such as potentially revealing important posttranscriptional regulatory events. Such differences represent an avenue for future work making reuse of the data that we provide.

In addition to being a rare disease, the fact that PPCM has onset in the peripartum period also hinders the collection of samples. Our myocardial samples were collected from tissue explanted during either heart transplantation or left ventricular assist device implantation: at the end-stage of the disease trajectory. Our patient population therefore reflects a selection bias for the most serious of cases. The fact that we observed analogous changes in blood serum samples collected shortly after parturition (*i*.*e*.*,* earlier in the disease progression) however lends credence to the hypothesis that chymase could be mechanistically involved in the development of the disease. However the changes that we observe cannot be assumed to generalize to patients with a more moderate phenotype or earlier in the development of the disease without additional evidence.

Due to our use of human samples rather than designed experiments, we cannot be sure that the increase in CPA3 and CMA1 is not related to any treatment received by patients with PPCM, and not present in both NPCM and control patients. Clinical guidelines suggest similar management of patients with PPCM and NPCM, and indeed the prevalence of medications for heart failure in our cohort did not differ between these groups.

## Conclusion

We utilized an unbiased approach, integrating data across multiple -omics modalities (proteomics, single nucleus transcriptomics, spatial transcriptomics) and validated our findings against transcriptomics and blood serum proteomics from independent cohorts. The mast cell proteases chymase and carboxypeptidase A3 are upregulated specifically in heart tissue in PPCM in contrast to NPCM and controls. We show that chymase negatively impacts cardiomyocyte phenotype, suggesting mechanistic relevance to pathophysiology. Furthermore, we demonstrate in proof-of-principle that CMA1 could be used as a biomarker for diagnosis of PPCM in peripartum women.

## Data Availability

The mass spectrometry proteomics data have been deposited to the ProteomeXchange Consortium via the PRIDE partner repository with the dataset identifier PXD061985. Due to GDPR regulations, sequencing data underlying the single nucleus and spatial transcriptomics cannot be made publicly available. These data can be accessed upon reasonable request to the corresponding author, subject to appropriate data sharing agreements and institutional approvals.

## Supplemental Data

This article contains supplemental data (25, 45).

## Conflict of Interest

The authors declare no competing interests.
