# Supplementary material for: An Unbiased Molecular Characterization of Peripartum Cardiomyopathy Hearts Identifies Mast Cell Chymase as a New Diagnostic Candidate
Source: Mol Cell Proteomics. 2026 Jan 13;25(2):101510. doi: 10.1016/j.mcpro.2026.101510 (PMC12906182; doi:10.1016/j.mcpro.2026.101510)
Supplement: Supplementary Material 2 [file mmc2.pdf]

Figure S1.

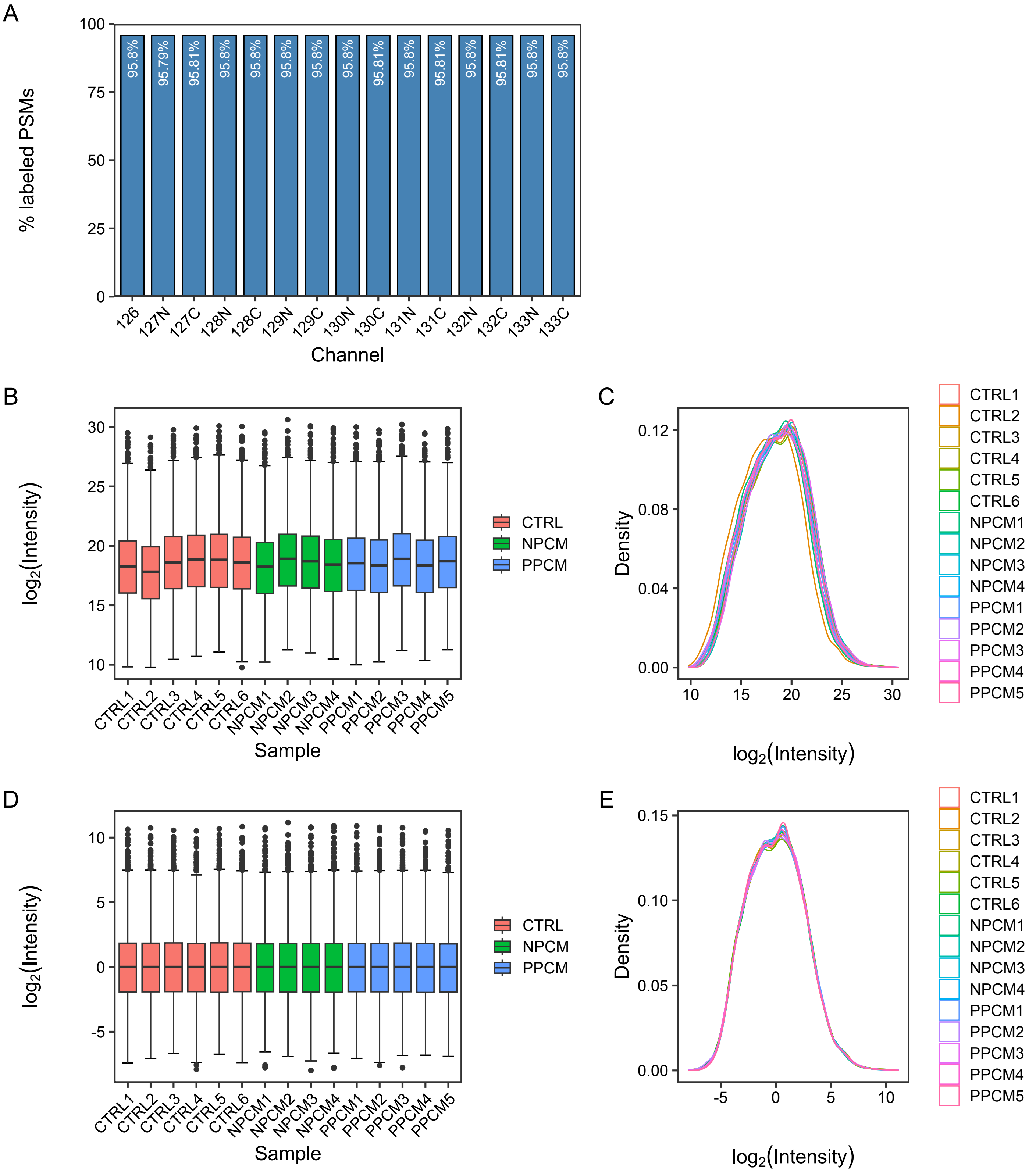
A
100
75
50
25
0
% labeled PSMs
95.8%
95.79%
95.81%
95.8%
95.8%
95.8%
95.8%
95.8%
95.81%
95.8%
95.81%
95.8%
95.81%
95.8%
95.8%
126
127N
127C
128N
128C
129N
129C
130N
130C
131N
131C
132N
132C
133N
133C
Channel
B
log2(Intensity)
CTRL1
CTRL2
CTRL3
CTRL4
CTRL5
CTRL6
NPCM1
NPCM2
NPCM3
NPCM4
PPCM1
PPCM2
PPCM3
PPCM4
PPCM5
Sample
CTRL
NPCM
PPCM
C
Density
log2(Intensity)
D
log2(Intensity)
Sample
E
Density
log2(Intensity)

**Supplementary Figure 1. Quality control of quantitative tissue proteomics. A.** Labelling efficiency of peptides with TMTpro reagents. Fraction of labelled peptides relative to all identified peptides, shown for each channel. **B-C.** $Log_2$ transformed isobaric protein quantifications per sample before normalisation. **D-E.** $Log_2$ transformed isobaric protein quantifications per sample after normalisation by median subtraction.

TMT - tandem mass tag

# Figure S2.

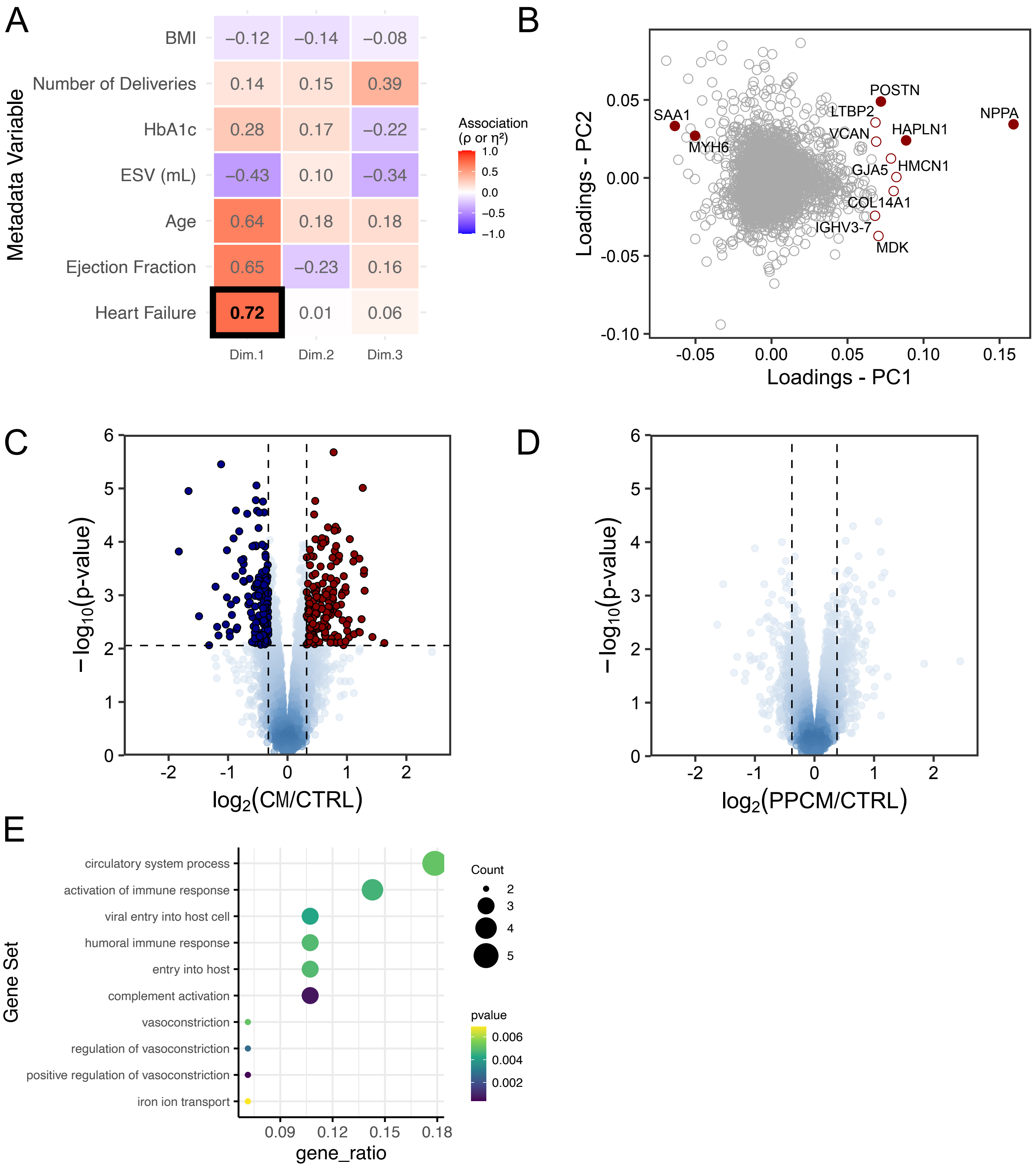
A
Metadata Variable
BMI
Number of Deliveries
HbA1c
ESV (mL)
Age
Ejection Fraction
Heart Failure
−0.12 −0.14 −0.08
0.14 0.15 0.39
0.28 0.17 −0.22
−0.43 0.10 −0.34
0.64 0.18 0.18
0.65 −0.23 0.16
0.72 0.01 0.06
Dim.1 Dim.2 Dim.3
Association (ρ or η²)
1.0 0.5 0.0 −0.5 −1.0
B
Loadings - PC2
Loadings - PC1
POSTN
LTBP2
SAA1
VCAN
HAPLN1
NPPA
MYH6
HMCN1
GJA5
COL14A1
IGHV3-7
MDK
C
−log10(p-value)
log2(CM/CTRL)
D
−log10(p-value)
log2(PPCM/CTRL)
E
Gene Set
circulatory system process
activation of immune response
viral entry into host cell
humoral immune response
entry into host
complement activation
vasoconstriction
regulation of vasoconstriction
positive regulation of vasoconstriction
iron ion transport
gene_ratio
0.09 0.12 0.15 0.18
Count
2 3 4 5
pvalue
0.006 0.004 0.002

**Supplementary Figure 2. Downstream analysis of tissue proteomes. A.** Association between principal components and clinical data. Principal components explaining >10% of variance were tested against potentially relevant clinical variables using Spearman correlation (continuous) or Kruskal-Wallis (categorical). Values show effect sizes (ρ or $\eta^2$); bold cells with black borders indicate statistical significance after multiple testing correction (adjusted $p < 0.05$). Heart failure status statistically associates with PC1, but no other clinical variables show significant associations**. B.** Loadings of proteins upon principal components 1 and 2, highlighting proteins contributing most of component 1: separating heart failure (of any aetiology) from control. **C.** 156 proteins were statistically upregulated and 116 downregulated between heart failure (of any aetiology) and control samples **D.** No proteins were statistically regulated between PPCM and control samples at a false discovery rate of 0.05. **E.** Overrepresented Gene Ontology biological processes among gene products which are statistically downregulated in PPCM compared to control in the MAGnet transcriptome, and change in the same direction in the tissue proteome of this study. The 10 gene sets with the smallest *p* value are shown.

# Figure S3.

A

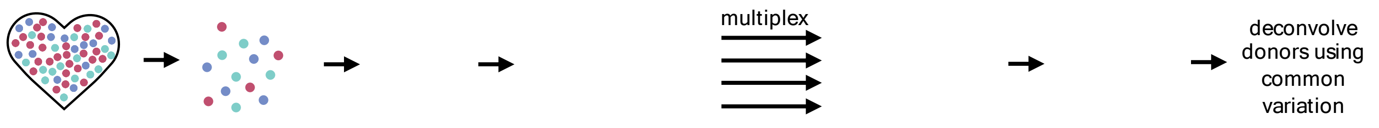


B

| Donor | RIN (AU) |
|---|---|
| PPCM1 | 8.2 |
| PPCM2 | 8.1 |
| PPCM3 | 7.2 |
| PPCM4 | 8.2 |
| PPCM5 | 8.5 |
| NPCM1 | 7.8 |
| NPCM2 | 8 |
| NPCM3 | 8.4 |
| NPCM4 | 8.3 |
| CTRL1 | 7.7 |
| CTRL2 | 8.3 |
| CTRL3 | 8.1 |
| CTRL4 | 8.9 |
| CTRL5 | 8.4 |
| CTRL6 | 8.8 |

C

D

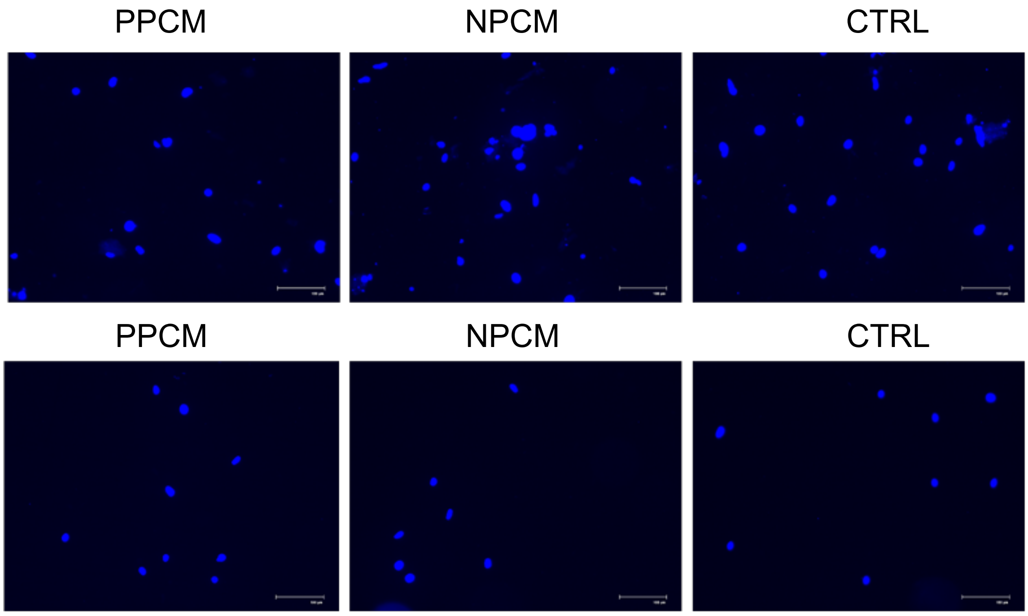


E

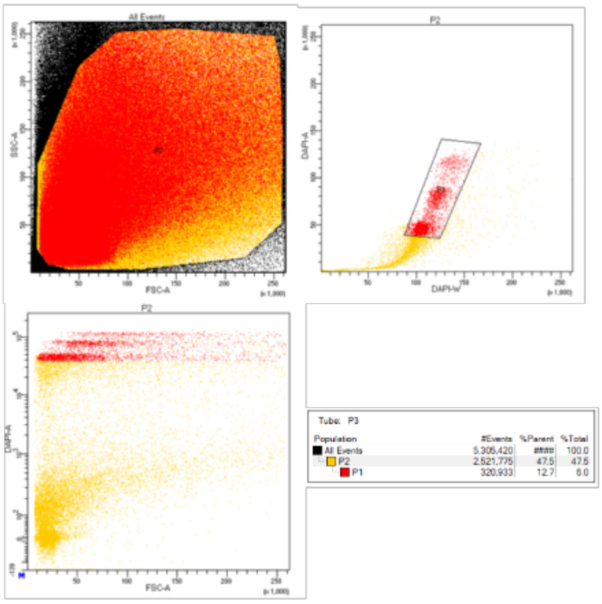


F

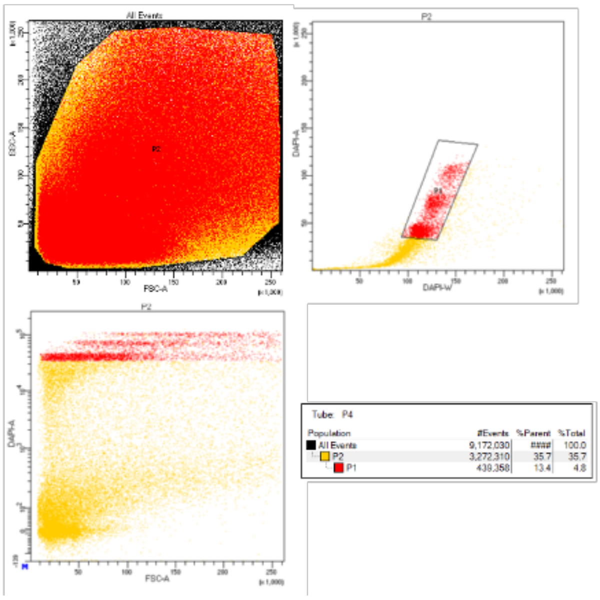


G

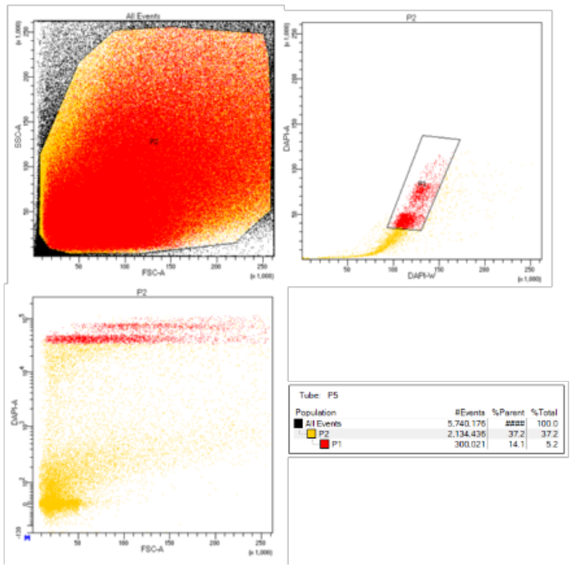

**Supplementary Figure 3. Single Nucleus RNAseq profiling of heart tissue: experimental overview. A.** Workflow for processing of samples for single nucleus RNA sequencing **B.** RNA quality (RIN - RNA integrity number) of heart tissue samples. **C.** Exemplar images of nuclei integrity and purity before FACS sorting, stained with NucBlue Live ready Probes. **D.** Nuclei integrity and purity assessed likewise after FACS sorting. **E-G**. FACS sorting of nuclei for pooled PPCM (**E**), NPCM (**F**) and non-failing control (**G**) groups respectively. Gating was applied to purify nuclei from the debris. Size gating was used to remove aggregates, multiplets or doublets was applied. Hoechst 33342 positive nuclei were selected in P2. Further gating for size was applied (P1) and single nuclei were sorted (red population).

RIN – RNA Integrity Number

# Figure S4.

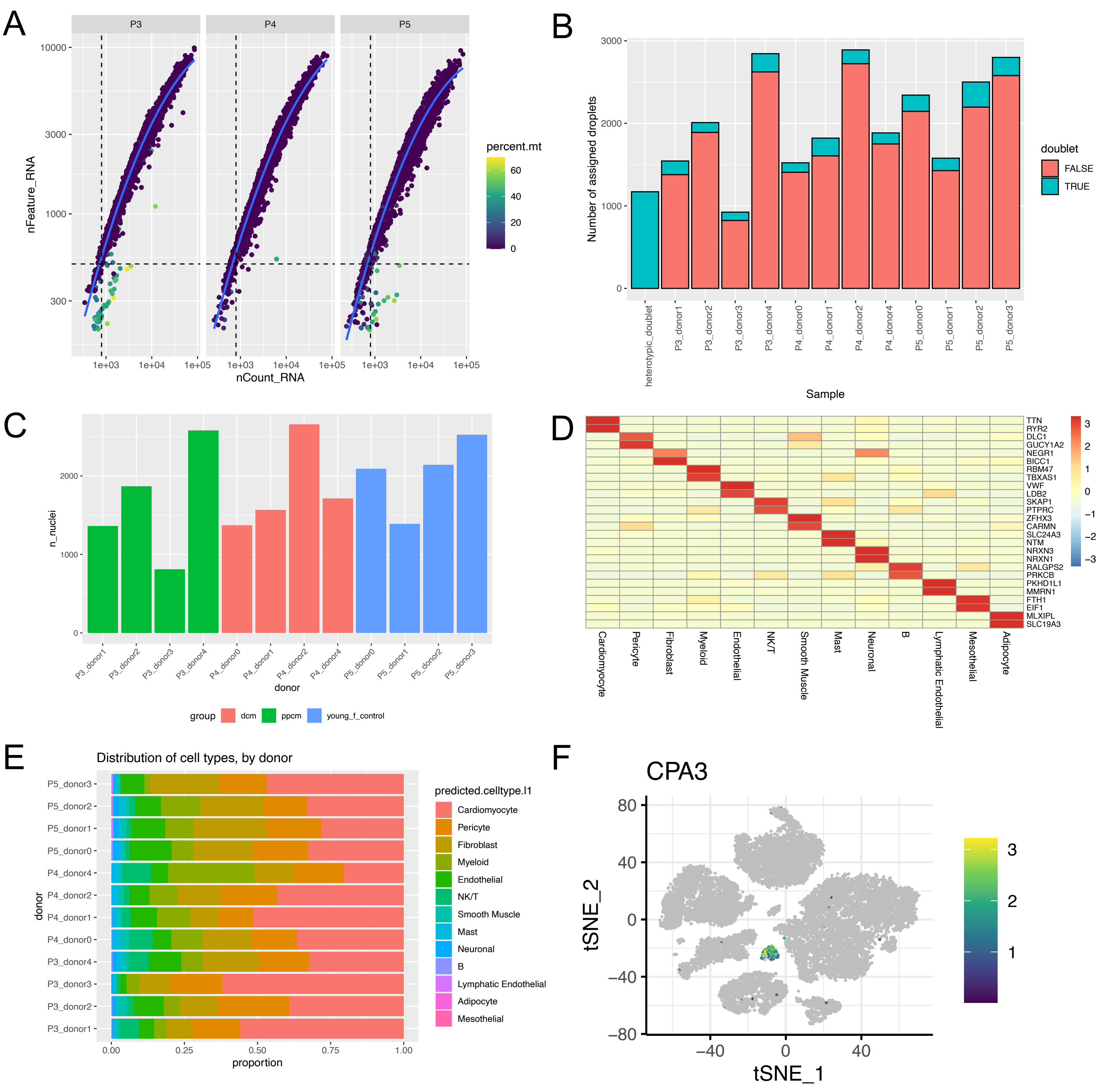
A
P3
P4
P5
nFeature_RNA
nCount_RNA
percent.mt
B
Number of assigned droplets
Sample
doublet
FALSE
TRUE
C
n_nuclei
donor
group
dcm
ppcm
young_f_control
D
Cardiomyocyte
Pericyte
Fibroblast
Myeloid
Endothelial
NK/T
Smooth Muscle
Mast
Neuronal
B
Lymphatic Endothelial
Mesothelial
Adipocyte
TTN
RYR2
DLC1
GUCY1A2
NEGR1
BICC1
RBM47
TBXAS1
VWF
LDB2
SKAP1
PTPRC
ZFHX3
CARMN
SLC24A3
NTM
NRXN3
NRXN1
RALGPS2
PRKCB
PKHD1L1
MMRN1
FTH1
EIF1
MLXIPL
SLC19A3
E
Distribution of cell types, by donor
donor
proportion
predicted.celltype.l1
F
CPA3
tSNE_1
tSNE_2

**Supplementary Figure 4. Single Nucleus RNAseq profiling of heart tissue: downstream analysis. A.** Quality control was based upon minimum unique molecular identifier count > 800, minimum number of genes > 500, maximum percentage of reads mapping to mitochondrial genes < 20 %. Multiplexed samples are shown here, corresponding to a single flowcell. **B.** An average of 12.5 % of droplets were assigned as doublets, using either the presence of genes from two different individuals (based on common genetic variants) to call heterotypic doublets or based upon gene expression profiles to call homotypic doublets. **C.** An average of 1,837 nuclei were measured per individual. **D.** Heatmap showing the z-scored expression, normalised for sequencing depth, for the top 2 marker genes per cell type. **E.** Cell type composition of the samples from each individual after reference free deconvolution using common genetic variation showed a large amount of heterogeneity. **F.** Neighbour embedding, showing that CPA3 expression is restricted to mast cells.

P3 = PPCM, P4 = NPCM, P5 = CTRL

# Figure S5.

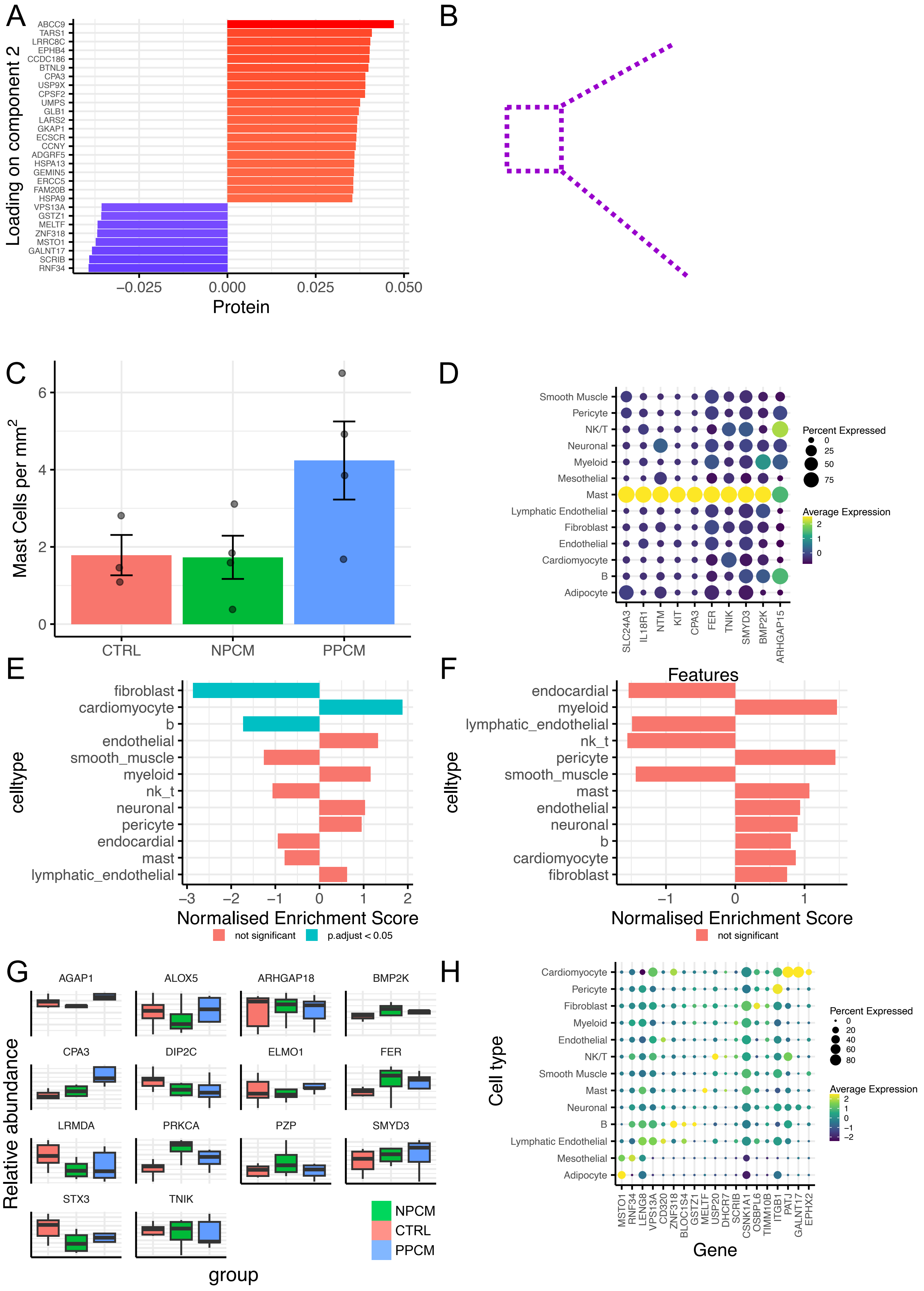
A
Loading on component 2
ABCC9
TARS1
LRRC8C
EPHB4
CCDC186
BTNL9
CPA3
USP9X
CPSF2
UMPS
GLB1
LARS2
GKAP1
ECSCR
CCNY
ADGRF5
HSPA13
GEMIN5
ERCC5
FAM20B
HSPA9
VPS13A
GSTZ1
MELTF
ZNF318
MSTO1
GALNT17
SCRIB
RNF34
−0.025
0.000
0.025
0.050
Protein
B
C
Mast Cells per mm2
CTRL
NPCM
PPCM
D
Smooth Muscle
Pericyte
NK/T
Neuronal
Myeloid
Mesothelial
Mast
Lymphatic Endothelial
Fibroblast
Endothelial
Cardiomyocyte
B
Adipocyte
SLC24A3
IL18R1
NTM
KIT
CPA3
FER
TNIK
SMYD3
BMP2K
ARHGAP15
Features
Percent Expressed
Average Expression
E
celltype
fibroblast
cardiomyocyte
b
endothelial
smooth_muscle
myeloid
nk_t
neuronal
pericyte
endocardial
mast
lymphatic_endothelial
Normalised Enrichment Score
not significant
p.adjust < 0.05
F
celltype
endocardial
myeloid
lymphatic_endothelial
nk_t
pericyte
smooth_muscle
mast
endothelial
neuronal
b
cardiomyocyte
fibroblast
Normalised Enrichment Score
not significant
G
AGAP1
ALOX5
ARHGAP18
BMP2K
CPA3
DIP2C
ELMO1
FER
LRMDA
PRKCA
PZP
SMYD3
STX3
TNIK
Relative abundance
group
NPCM
CTRL
PPCM
H
Cell type
Cardiomyocyte
Pericyte
Fibroblast
Myeloid
Endothelial
NK/T
Smooth Muscle
Mast
Neuronal
B
Lymphatic Endothelial
Mesothelial
Adipocyte
MSTO1
RNF34
LENG8
VPS13A
CD320
ZNF318
BLOC1S4
GSTZ1
MELTF
USP20
DHCR7
SCRIB
CSNK1A1
OSBPL6
TIMM10B
ITGB1
PATJ
GALNT17
EPHX2
Gene
Percent Expressed
Average Expression

**Supplementary Figure 5. Partial least square discriminant analysis suggests an involvement of mast cells proteases CMA1 and CPA3 in PPCM. A.** Top 50 proteins contributing the most to the separation of PPCM samples from both the control and NPCM groups along component 2. **B.** Immunohistochemistry using an antibody for tryptase, a canonical marker of mast cells. **C.** There is no evidence that PPCM samples contain a higher density of tryptase positive mast cells compared to either controls or NPCM (ANOVA without assuming equal variance, $p = 0.1103$). **D.** Expression across all identified cell types of the 10 genes whose expression is most specific to mast cells, calculated via the area under a receiver operating characteristic for the ability of that gene to classify a given cell as a mast cell or other cell type. **E.** Enrichment of cell types was calculated upon component 1 using sets of genes that are predominantly expressed in particular cell types in the single nucleus RNAseq data of (45).The enrichment along component 1 shows the expected changes in end stage heart failure, with an enrichment of cardiomyocyte proteins in controls compared to heart failure of either aetiology, and an enrichment of fibroblast and B cell derived proteins in heart failure. **F.** There was no enrichment of any particular cell type upon component 2 (separating PPCM samples from both other groups) – indicating that there is no evidence for a general increase or decrease in any cell type. **G.** Abundance of proteins whose expression is specific to mast cells by disease status, in the heart cell atlas (25). **H.** Cell type expression profiles derived from snRNAseq of the top 50 proteins contributing to the separation of PPCM along component 2 of the PLS-DA upon the proteomics and are negatively associated with PPCM.

Figure S6.

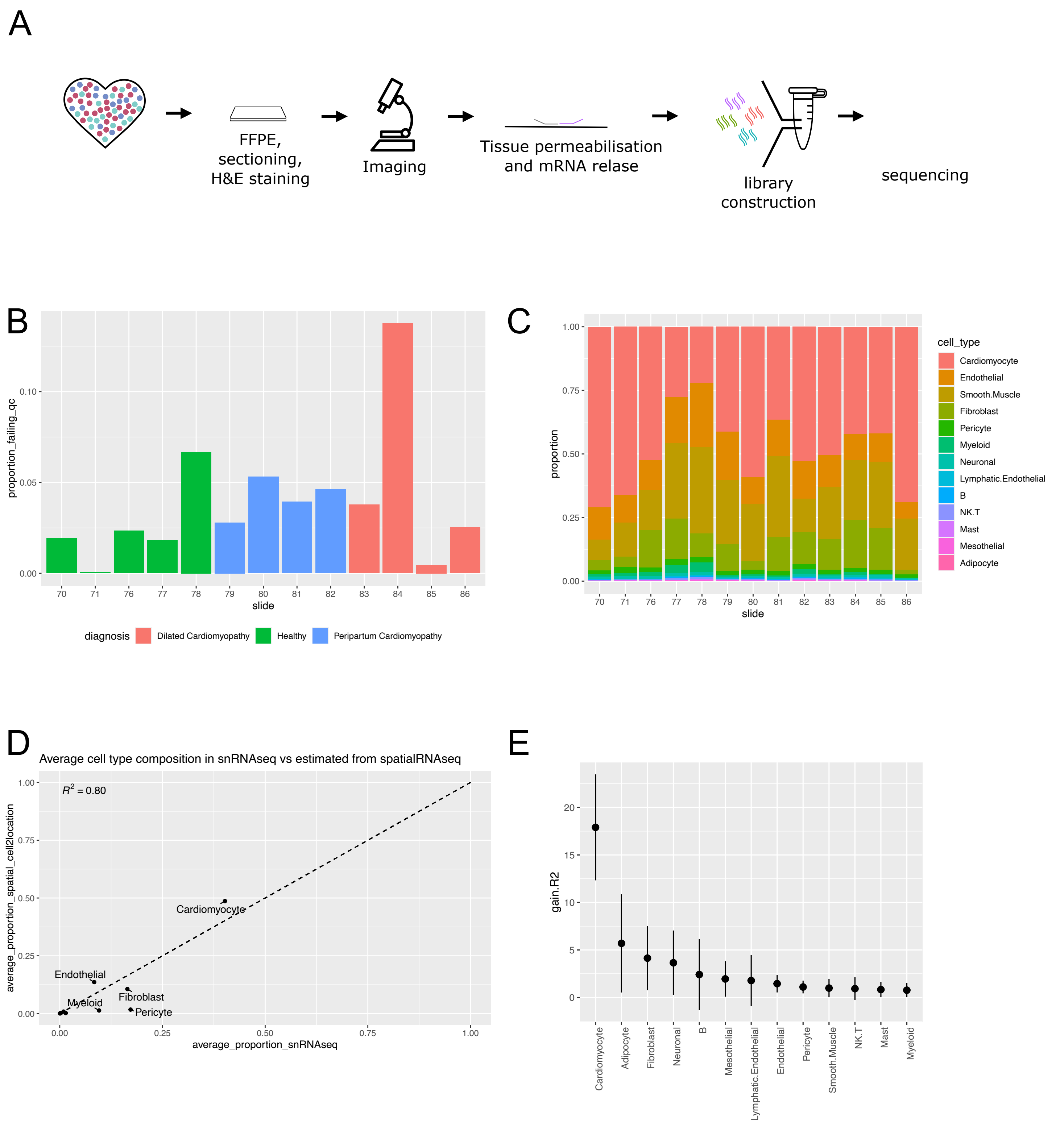
A
FFPE,
sectioning,
H&E staining
Imaging
Tissue permeabilisation
and mRNA relase
library
construction
sequencing
B
proportion_failing_qc
0.10
0.05
0.00
70
71
76
77
78
79
80
81
82
83
84
85
86
slide
diagnosis
Dilated Cardiomyopathy
Healthy
Peripartum Cardiomyopathy
C
proportion
1.00
0.75
0.50
0.25
0.00
70
71
76
77
78
79
80
81
82
83
84
85
86
slide
cell_type
Cardiomyocyte
Endothelial
Smooth.Muscle
Fibroblast
Pericyte
Myeloid
Neuronal
Lymphatic.Endothelial
B
NK.T
Mast
Mesothelial
Adipocyte
D
Average cell type composition in snRNAseq vs estimated from spatialRNAseq
average_proportion_spatial_cell2location
$R^2 = 0.80$
1.00
0.75
0.50
0.25
0.00
Cardiomyocyte
Endothelial
Fibroblast
Myeloid
Pericyte
0.00
0.25
0.50
0.75
1.00
average_proportion_snRNAseq
E
gain.R2
20
15
10
5
0
Cardiomyocyte
Adipocyte
Fibroblast
Neuronal
B
Mesothelial
Lymphatic.Endothelial
Endothelial
Pericyte
Smooth.Muscle
NK.T
Mast
Myeloid

**Supplementary Figure 6. Spatial transcriptomics profiling of heart tissue. A.** Frozen tissue sections were directly formalin fixed and paraffin embedded, and then processed according to manufacturer workflows for Visium spatial transcriptomics (10x Genomics, USA). An H&E stained image of the tissue is collected, before tissue is permeabilised to allow probes with whole genome coverage local access to endogenous RNA. This is captured upon spots in the slide, before synthesis of cDNA which incorporates spatial barcodes. Library preparation and sequencing proceed analogous to conventional RNAseq workflows. **B.** A mean of 3.8 % of spots over tissue on the spatial transcriptomics array failed the quality control criteria. **C.** Estimated cell type abundance in samples from each individual. **D.** Correlation between average estimated cell type abundance across all samples derived from the deconvolved spatial transcriptome and cell type abundance observed in single nucleus RNAseq ($R^2$ = 0.80, $p$ = 0.0004). **E.** Gain in predictive performance of multiview model to predict the abundance of cell types by the presence of other cell types. The gain in $R^2$ describes the additional proportion of variance explained when including the local neighbours and broader tissue structure in addition to the immediately co-located cells (i.e. within the same spot). Mast cells showed only a very small increase when accounting for these additional tissue structures.

# Figure S7

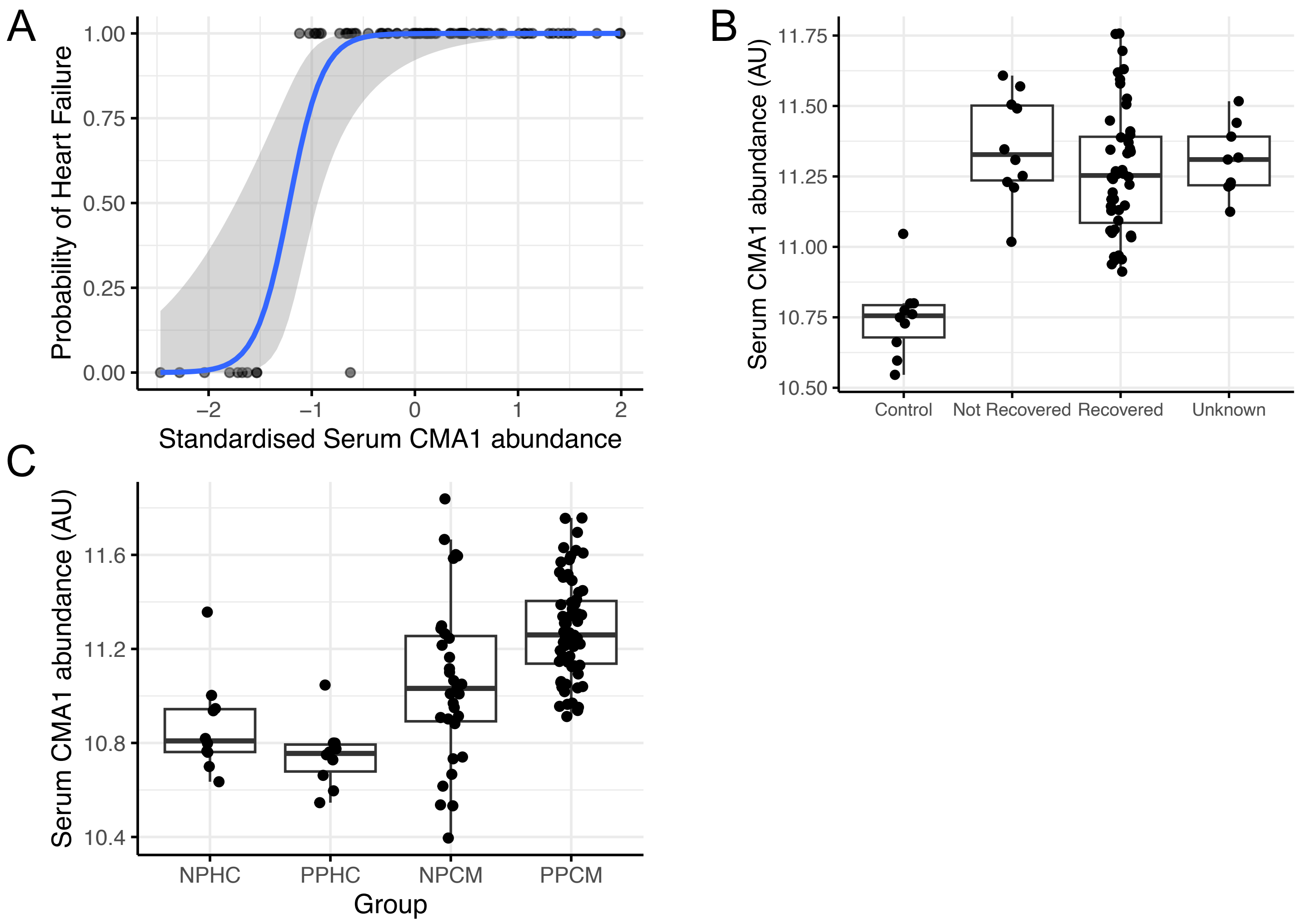

A
Probability of Heart Failure
1.00
0.75
0.50
0.25
0.00
−2
−1
0
1
2
Standardised Serum CMA1 abundance
B
Serum CMA1 abundance (AU)
11.75
11.50
11.25
11.00
10.75
10.50
Control
Not Recovered
Recovered
Unknown
C
Serum CMA1 abundance (AU)
11.6
11.2
10.8
10.4
NPHC
PPHC
NPCM
PPCM
Group

**Supplementary Figure 7. Chymase in peripheral blood serum classifies PPCM in peripartum women. A.** Logistic regression model to predict PPCM in peripartum individuals utilising CMA1 abundance the peripheral blood serum proteome. For a one standard deviation increase in CMA1 abundance, there is an increase in the odds ratio for heart failure of 474. **B.** CMA1 abundance in PPCM patients is not informative of patient recovery within the first 12 months following diagnosis (*F*-test between PPCM patients grouped by recovery status at 12 months, $p$ = 0.268). C. Mean CMA1 abundance is elevated in the serum of PPCM compared to NPCM (Welch's t-test, p = 0.002), but with limited discriminative performance (AUROC = 0.70).
